# Holdfast-Derived Lipid Extract from the Patagonian Macroalga *Macrocystis pyrifera* Attenuates Amyloid-β-Induced Neuronal Metabolic Dysfunction In Vitro and Ex Vivo

**DOI:** 10.3390/ph19091489

**Published:** 2026-09-18

**Authors:** Fabio Méndez, Pedro Cisternas, Paulina Ormazabal, Camila Gherardelli, Máximo Frangopulos, Andrés Mansilla, Nibaldo C. Inestrosa

**Affiliations:** 1 Laboratorio de Ecosistemas Marinos Antárticos y Subantárticos (LEMAS), Facultad de Ciencias, Universidad de Magallanes, Punta Arenas 6210427, Chile; fabiomendezmansilla@gmail.com (F.M.); andres.mansilla@umag.cl (A.M.); 2 Centro de Excelencia en Biomedicina de Magallanes (CEBIMA), Escuela de Medicina, Universidad de Magallanes, Punta Arenas 6210427, Chile; pcisternasf@udla.cl; 3 Núcleo de Investigación en Nutrición y Ciencias Alimentarias (NINCAL), Facultad de Salud y Ciencias Sociales, Universidad de las Américas, Santiago 7500658, Chile; 4 Facultad de Ciencias para el Cuidado de la Salud, Universidad San Sebastián, Lota 2465, Providencia, Santiago 7510157, Chile; paulina.ormazabal@uss.cl; 5 Mitchell Center for Alzheimer’s Disease and Related Brain Disorders, Department of Neurology, University of Texas McGovern Medical School at Houston, Houston, TX 77030, USA; cgherardelli@gmail.com; 6 Centro de Investigación GAIA-Antártica (CIGA), Universidad de Magallanes, Punta Arenas 6210427, Chile; max.frangopulos@umag.cl

**Keywords:** Alzheimer’s disease, neuronal glucose metabolism, amyloid-β, *Macrocystis pyrifera*, marine lipid extract

## Abstract

**Background**: Impaired cerebral glucose metabolism is an early and clinically relevant feature of Alzheimer’s disease (AD). Amyloid-β (Aβ) accumulation can further disrupt neuronal energy homeostasis, increasing metabolic and oxidative vulnerability. **Objective**: This study evaluated whether lipid extracts obtained from different morphological structures of the Patagonian macroalga could preserve neuronal glucose metabolism and viability under basal conditions and following exposure to an Aβ_1–42_ preparation generated under established oligomer-forming conditions. **Methods**: Lipid extracts derived from fronds (LEFF), stipes (LEFS), and holdfasts (LEFH) were applied at an equivalent total-lipid concentration of 0.1 µg/mL and evaluated in primary mouse hippocampal neurons and acute hippocampal slices. Neuronal viability, glucose uptake kinetics, glycolytic flux, pentose phosphate pathway activity, ATP/ADP ratio, AMPKα Thr172 phosphorylation, glutathione-dependent antioxidant parameters, and gene expression were assessed. **Results**: Among the extracts examined at the selected non-cytotoxic concentration of 0.1 µg/mL, the holdfast-derived lipid extract (LEFH) produced the largest biological effects. LEFH attenuated Aβ-induced neuronal death and preserved glucose uptake, glycolytic flux, pentose phosphate pathway activity, and the ATP/ADP ratio in Aβ-treated neurons. Kinetic analysis showed a significant overall difference in the apparent maximal glucose uptake rate among preparations (*p* = 0.019), with LEFH displaying the highest value, whereas the apparent Michaelis–Menten constant did not differ significantly among groups. LEFH also increased AMPKα Thr172 phosphorylation and Ppargc1a mRNA expression, supporting the engagement of an AMPK-associated metabolic response. In addition, LEFH increased intracellular glutathione content and glutathione peroxidase activity under basal conditions and selectively reduced Il6 mRNA expression, whereas Tnf mRNA remained unchanged. The principal effects on glucose uptake and cellular energy status were reproduced in Aβ-exposed hippocampal slices. **Conclusions**: These findings identify the LEFH of *M. pyrifera* as a bioactive marine preparation capable of attenuating Aβ-associated neuronal metabolic dysfunction. The results support a coordinated response involving glucose utilization, energy sensing, basal glutathione-associated parameters, and metabolic gene expression. Further chemical characterization, mechanistic validation, and chronic in vivo studies are required to identify the active components and establish their translational relevance.

## 1. Introduction

Glucose is the principal energy substrate of the mammalian brain and is essential for maintaining normal cerebral function [1,2,3]. Although the adult human brain represents only approximately 2% of total body weight, it accounts for nearly 20% of the body’s energy expenditure [4,5,6]. Glucose metabolism provides the ATP required to sustain essential neuronal processes, including membrane polarization, neurotransmission, and synaptic activity [3,7]. Consequently, alterations in glucose transport or utilization can disrupt cerebral energy homeostasis and increase neuronal vulnerability, a condition associated with several neurodegenerative disorders, including Alzheimer’s disease (AD) [8,9,10].

AD is a progressive, age-related neurodegenerative disorder characterized by cognitive decline and pathological alterations that include the extracellular accumulation of amyloid-β (Aβ) peptides in amyloid plaques and the intracellular deposition of hyperphosphorylated tau in neurofibrillary tangles [11,12,13]. These classical hallmarks are accompanied by oxidative stress, mitochondrial dysfunction, neuroinflammation, and reduced cerebral glucose utilization [13,14,15,16]. Reduced glucose metabolism, assessed through positron emission tomography (PET), has therefore emerged as a relevant indicator of neuronal dysfunction and disease progression. This metabolic decline affects brain regions that are particularly important for memory and cognition, including the hippocampus and cortical areas [13,17,18,19]. PET studies using radiolabeled glucose analogs have consistently demonstrated a progressive reduction in cerebral glucose utilization that parallels cognitive and clinical decline [8,20,21,22]. Importantly, cerebral hypometabolism may be detected during early disease stages and, in some individuals, several years before the appearance of overt clinical symptoms. Aβ accumulation can further compromise neuronal energy homeostasis through several complementary mechanisms. Aβ peptides interfere with glucose transporter expression and function, disrupt insulin-associated signaling, and impair glycolytic processes, ultimately limiting ATP availability [23,24].

Mitochondrial alterations contribute to this metabolic imbalance, as Aβ can disrupt electron transport chain activity, increase reactive oxygen species production, and affect mitochondrial dynamics [25,26,27,28,29]. Importantly, glycolytic and mitochondrial abnormalities have also been demonstrated across complementary experimental models of AD. In 5xFAD mice, early amyloid pathology is accompanied by reduced hippocampal tricarboxylic acid cycle metabolism and selective impairment of both synaptic mitochondrial and glycolytic function, together with alterations in mitochondrial ultrastructure. In contrast, cortical oxidative glucose metabolism may increase, suggesting region-specific compensatory responses [30]. In the same model, progressive alterations in mitochondrial biogenesis, fission and fusion, mitophagy, oxygen consumption, ATP generation, and Ca^2+^ handling have been detected as early as 2 months of age, supporting the concept that mitochondrial dysfunction can emerge during early amyloid pathology [31]. Consistent with this metabolic heterogeneity, neurons and astrocytes derived from induced pluripotent stem cells from individuals with sporadic AD exhibit reduced mitochondrial respiration accompanied by increased glycolytic flux and alterations in mitochondrial dynamics [32]. These findings indicate that AD-related bioenergetic dysfunction is not restricted to a single metabolic pathway but involves coordinated, region-, cell-, and model-dependent changes in glycolysis and mitochondrial function. Such loss of metabolic flexibility may compromise the capacity of neurons to meet energetic demands and increase their vulnerability to amyloidogenic stress.

The lipid fraction was selected because macroalgal lipids comprise a chemically and biologically relevant pool of fatty acids, sterols, and other lipophilic metabolites with the potential to modulate cellular metabolism and neurodegeneration-related pathways. Polyunsaturated fatty acids and other lipid-associated compounds derived from marine algae have been linked to antioxidant, anti-inflammatory, and neuroprotective effects in experimental systems [33]. More specifically, lipid extracts from brown seaweeds have been shown to activate liver X receptors (LXRs) and peroxisome proliferator-activated receptors (PPARs), thereby modulating transcriptional programs involved in lipid and cholesterol metabolism and inflammation [34]. Importantly, recent in vivo evidence further supports the biological relevance of this fraction: dietary supplementation with a lipid extract from the brown seaweed *Himanthalia elongata* attenuated cognitive deterioration and reduced neuroinflammation-associated markers in APPswePS1ΔE9 mice [35]. Based on this evidence, and on the distinct fatty-acid profiles observed among the fronds, stipes, and holdfasts of *M. pyrifera*, we focused specifically on the lipid fraction to determine whether this anatomical chemical heterogeneity translated into differential effects on neuronal glucose metabolism and resistance to Aβ-associated stress.

*Macrocystis pyrifera* is a foundation-forming giant kelp and an abundant component of sub-Antarctic coastal ecosystems in southern Patagonia [36,37,38]. The Strait of Magellan supports extensive *M. pyrifera* forests, where this species acts as a major habitat-forming component and contributes substantially to local ecosystem structure and biodiversity [39]. Recent studies conducted at multiple sites along the Strait of Magellan further confirm the broad regional occurrence of *M. pyrifera* across environmentally contrasting sectors and emphasize its ecological importance as a foundational species [38,40]. The present investigation was conceived within a regional bioprospecting framework aimed at exploring abundant Patagonian marine resources as potential sources of bioactive compounds. *M. pyrifera* was selected because of its regional availability and ecological relevance, together with its pronounced morphological differentiation into fronds, stipes, and holdfasts. This anatomical heterogeneity provided a natural basis for comparing structure-derived lipid extracts and identifying preparations with distinct metabolic and neuroprotective activity.

We hypothesized that lipid extracts derived from fronds (LEFF), stipes (LEFS), and holdfasts (LEFH) of *M. pyrifera* would differentially modulate neuronal metabolism and that at least one of these preparations would attenuate Aβ-associated metabolic dysfunction. To test this hypothesis, lipid extracts derived from fronds, stipes, and holdfasts were evaluated in primary mouse hippocampal neurons and acute hippocampal slices under basal conditions and following exposure to an Aβ_1–42_ preparation generated using an established oligomer-forming protocol. Neuronal viability, glucose uptake and utilization, cellular energy status, glutathione-dependent antioxidant responses, AMPKα phosphorylation, and the expression of genes associated with glucose transport, lipid metabolism, inflammation, and neuronal signaling were examined. The frond-, stipe-, and holdfast-derived lipid extracts are hereafter designated LEFF, LEFS, and LEFH, respectively.

## 2. Results

### 2.1. Lipid Composition Differs Among the Morphological Structures of M. pyrifera

**Figure 1:**
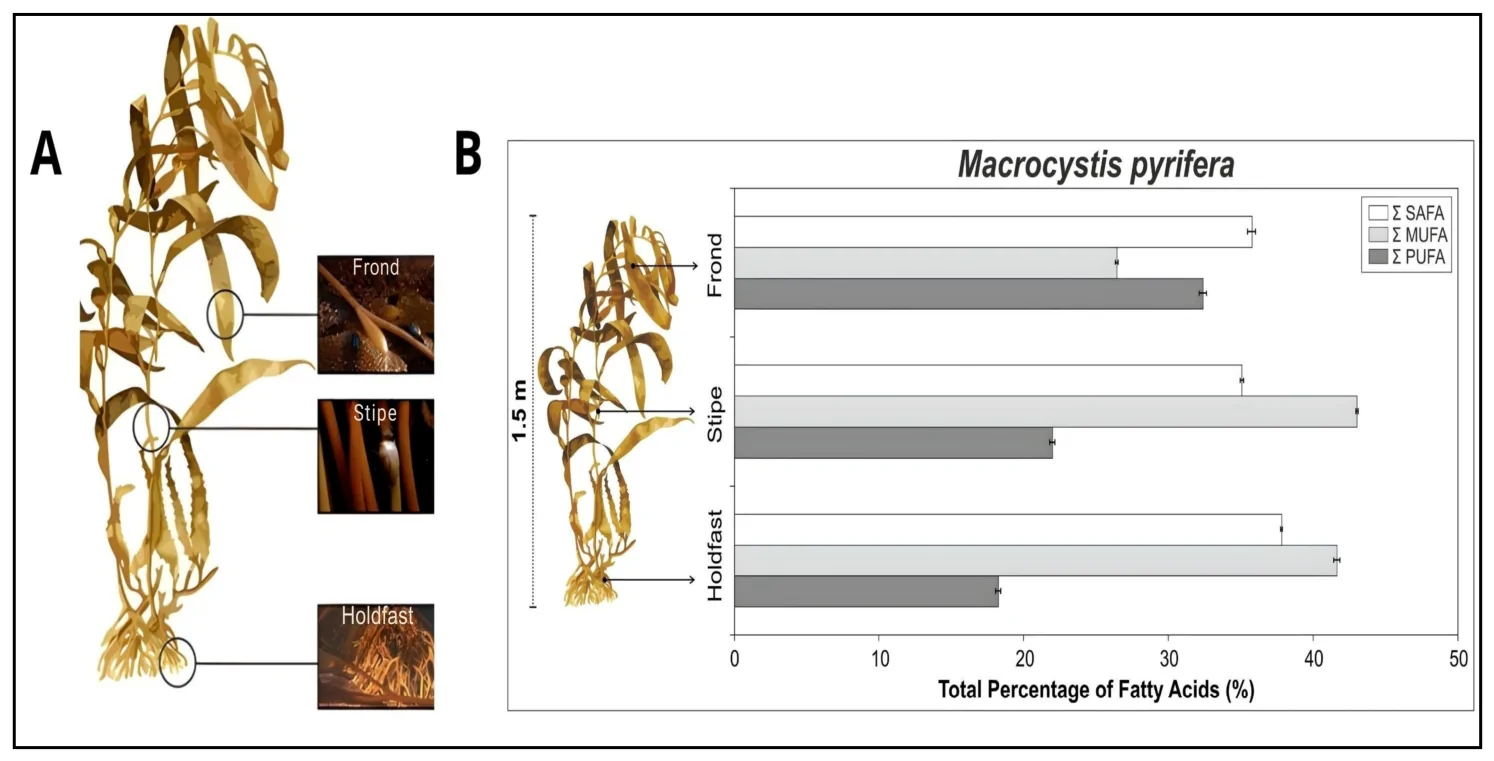
Morphological structures and fatty-acid class distribution in *Macrocystis pyrifera*. (**A**) Representative specimen of *M. pyrifera* showing the three anatomical structures analyzed: frond, stipe, and holdfast. Insets show representative images of each structure. The specimen illustration and photographic insets in panel (**A**) were produced by the authors and have not been previously published. (**B**) Relative abundance of saturated fatty acids (SAFA), monounsaturated fatty acids (MUFA), and polyunsaturated fatty acids (PUFA) in lipid extracts obtained separately from fronds, stipes, and holdfasts. Fatty-acid classes are expressed as percentages of the total identified fatty acids and presented as mean ± SD from three independent extractions per anatomical structure. The numerical fatty-acid data were reanalyzed from the GC-FID dataset previously reported by Méndez et al. [36]. Panel (**B**) was newly plotted from those numerical data for the present manuscript and does not reproduce a previously published graphical figure.

The fronds, stipes, and holdfasts of *M. pyrifera* were processed separately to compare the lipid composition of the three morphological structures represented (Figure 1A). Gravimetric analysis revealed differences in total lipid yield, with the highest lipid content detected in the stipes, followed by the fronds and holdfasts.

The distribution of the main fatty acid classes also varied among structures. Saturated fatty acids represented a similar proportion of the profiles obtained from fronds, stipes, and holdfasts. By contrast, monounsaturated fatty acids were more abundant in the stipe and holdfast extracts than in the frond extract, whereas the fronds contained the highest proportion of polyunsaturated fatty acids (Figure 1B). Seventeen individual fatty acids were detected across the three anatomical structures. Among them, C14:0, C16:0, C18:1n-9c, C20:5n-3, and C22:1n-9 each accounted for at least 10% of the fatty-acid profile in one or more morphological structures (Supplementary Table S1). These compositional differences provided the basis for comparing the biological activity of the frond-, stipe-, and holdfast-derived lipid extracts. The fatty-acid profiles obtained from the three independent extractions per anatomical structure are summarized in Supplementary Table S1 as mean ± SD. The fatty-acid data presented in Figure 1B and Supplementary Table S1 were reanalyzed from the GC-FID dataset previously reported by Méndez et al. [36]. They derive from the same collection and extraction series as the lipid preparations used in the biological assays. Figure 1B and Supplementary Table S1 were newly generated for the present manuscript.

### 2.2. The Holdfast Lipid Extract Attenuates Aβ_1–42_-Associated Neuronal Loss and Differentially Affects Glucose Uptake

**Figure 2:**
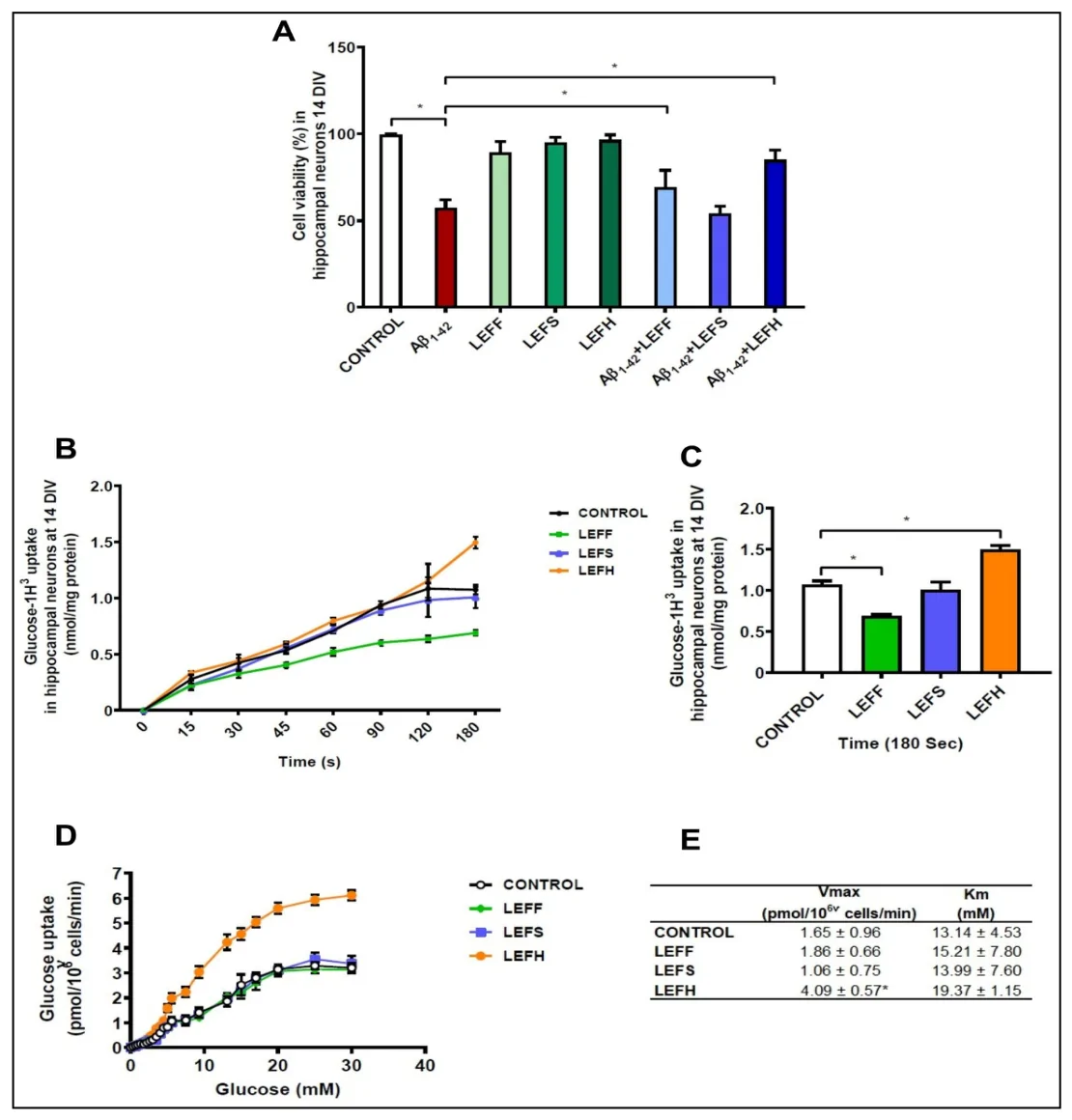
Lipid extracts from distinct *M. pyrifera* structures differentially affect neuronal viability and glucose uptake. (**A**) Viability of primary mouse hippocampal neurons maintained for 14 DIV and treated with the LEFF, LEFS, or LEFH, in the absence or presence of Aβ_1–42_ preparation (5 µM, 5 h). Neuronal viability was assessed by Hoechst nuclear staining; cells without nuclear condensation or fragmentation were considered viable. (**B**) Time course of [^3^H]-2-deoxy-D-glucose uptake over 180 s in untreated neurons and cultures treated with LEFF, LEFS, or LEFH. (**C**) Glucose uptake quantified at the 180 s endpoint. (**D**) Observed concentration-dependent glucose-uptake values; connecting lines are visual guides and do not represent fitted curves. (**E**) Mean ± SD apparent Vmax and Km obtained by fitting each independent dataset separately to the Michaelis-Menten equation. Therefore, observed values in panel (**D**) may exceed the mean Vmax in panel (**E**). In panel (**E**), * *p* = 0.022 for LEFS versus LEFH. The kinetic analysis comprised 11 independent datasets yielding valid fits across the four groups. For panel (**A**), data are presented as mean ± SD from five independent neuronal culture preparations. Panels (**B**,**C**) represent four independent neuronal culture preparations. Panel (**A**) was analyzed by one-way ANOVA across the experimental treatment groups. Panel (**B**) was analyzed by two-way ANOVA considering treatment and time as factors, including the treatment × time interaction. Panel (**C**) and the kinetic parameters presented in panel (**E**) were analyzed by one-way ANOVA. Significant omnibus effects were followed by Bonferroni-adjusted multiple comparisons. Horizontal brackets indicate the groups compared; * *p* < 0.05. All lipid extracts were applied at 0.1 µg/mL of total lipid. CONTROL denotes vehicle-treated cultures receiving 0.03% (*v*/*v*) DMSO in PBS.

The effects of the lipid extracts on neuronal viability were first evaluated in primary hippocampal cultures exposed to Aβ_1–42_ preparation. Aβ_1–42_ reduced neuronal viability by approximately 40% relative to the untreated control. None of the extracts produced detectable cytotoxicity when administered alone. Both frond-derived lipid extract (LEFF) and LEFH significantly increased neuronal viability relative to neurons treated with Aβ_1–42_ alone (*p* = 0.039 and *p* < 0.001, respectively), whereas stipe-derived lipid extract (LEFS) did not. The protection conferred by LEFH was the most pronounced and restored viability to values indistinguishable from untreated controls (*p* = 1.000 versus control), whereas viability in the Aβ_1–42_ + LEFF condition remained intermediate (Figure 2A; Supplementary Table S2).

We next examined the acute effects of the extracts on neuronal glucose uptake. Time-course measurements showed that glucose incorporation increased progressively in control neurons and in cultures treated with LEFS or LEFH. In contrast, LEFF reduced glucose uptake throughout the assay (Figure 2B). Quantification at 180 s confirmed a reduction of approximately 40% in LEFF-treated neurons, whereas LEFH significantly increased glucose uptake above control levels. LEFS did not produce a significant change at this time point (Figure 2C).

To determine whether these changes reflected altered glucose transport kinetics, uptake was measured across increasing glucose concentrations (Figure 2D). Control neurons showed an apparent Vmax of 1.65 ± 0.96 pmol/10^6^ cells/min and an apparent Km of 13.14 ± 4.54 mM. LEFH displayed the highest apparent Vmax (4.09 ± 0.57 pmol/10^6^ cells/min), and the overall comparison among groups was significant (one-way ANOVA, *p* = 0.019; Supplementary Table S2). In Bonferroni-adjusted pairwise comparisons, LEFH differed significantly from LEFS (*p* = 0.022), whereas the comparison between LEFH and control did not reach statistical significance (*p* = 0.063; Supplementary Table S3). The apparent Km in LEFH-treated neurons was 19.37 ± 1.15 mM and did not differ significantly among groups (*p* = 0.737). Thus, LEFH was associated with a higher apparent maximal glucose uptake capacity, without evidence of altered apparent glucose affinity.

### 2.3. LEFH Attenuates Aβ-Induced Disruption of Neuronal Glucose Metabolism

**Figure 3:**
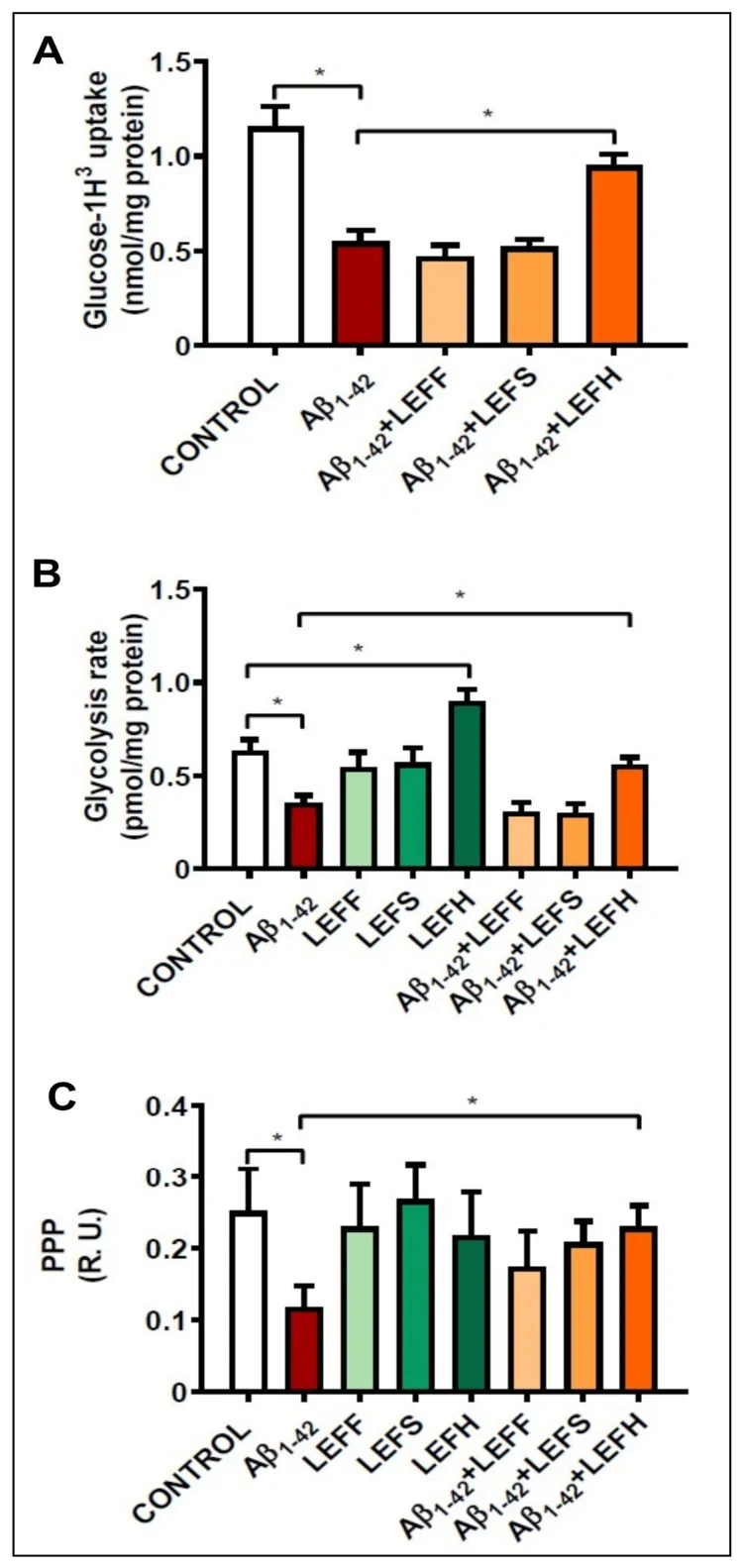
LEFH preserves neuronal glucose uptake and utilization following Aβ_1–42_ exposure. (**A**) [^3^H]-2-deoxy-D-glucose uptake in primary hippocampal neurons under control conditions or following exposure to Aβ_1–42_ preparation alone or after pretreatment with LEFF, LEFS, or LEFH. (**B**) Glycolytic flux determined by measuring ^3^H_2_O production from [3-^3^H]-glucose in neurons treated with the lipid extracts in the absence or presence of Aβ_1–42_. (**C**) Glucose oxidation through the pentose phosphate pathway under the same experimental conditions. Aβ_1–42_ reduced glucose uptake, glycolytic flux, and PPP-dependent glucose oxidation, whereas LEFH attenuated these metabolic alterations. Data are presented as mean ± SD from five independent neuronal culture preparations, with each condition analyzed in technical triplicate. Technical replicates were averaged before statistical analysis. Panels (**A**–**C**) were analyzed by one-way ANOVA across the corresponding experimental treatment groups. Significant omnibus effects were followed by Bonferroni-adjusted multiple comparisons. Horizontal brackets indicate the groups compared; * *p* < 0.05. All lipid extracts were applied at 0.1 µg/mL of total lipid. CONTROL denotes vehicle-treated cultures receiving 0.03% (*v*/*v*) DMSO in PBS.

Aβ_1–42_ exposure reduced neuronal glucose uptake by approximately 50% compared with control cultures. Pretreatment with LEFF or LEFS did not prevent this reduction. By contrast, LEFH significantly increased glucose uptake in Aβ-exposed neurons, maintaining values close to those observed under control conditions (Figure 3A).

A similar pattern emerged when intracellular glucose utilization was examined. The glycolytic rate decreased from 0.63 ± 0.06 pmol/mg protein under control conditions to 0.35 ± 0.04 pmol/mg protein following Aβ_1–42_ exposure. LEFF and LEFS did not prevent this decline. LEFH, however, significantly attenuated the effect of Aβ and maintained glycolytic activity near the control range (Figure 3B).

Glucose oxidation through the PPP was also impaired by Aβ_1–42_. The three lipid extracts did not markedly alter basal PPP activity, although LEFH produced the strongest response in Aβ-treated cultures. Neurons receiving LEFH before Aβ exposure showed significantly higher PPP-dependent glucose oxidation than neurons treated with Aβ alone, reaching values close to those of untreated cultures (Figure 3C). Together, these findings indicate that the protective action of LEFH extends beyond glucose entry and includes preservation of its utilization through both glycolysis and the PPP.

### 2.4. LEFH Increases Basal Neuronal GSH Content and GPx Activity

**Figure 4:**
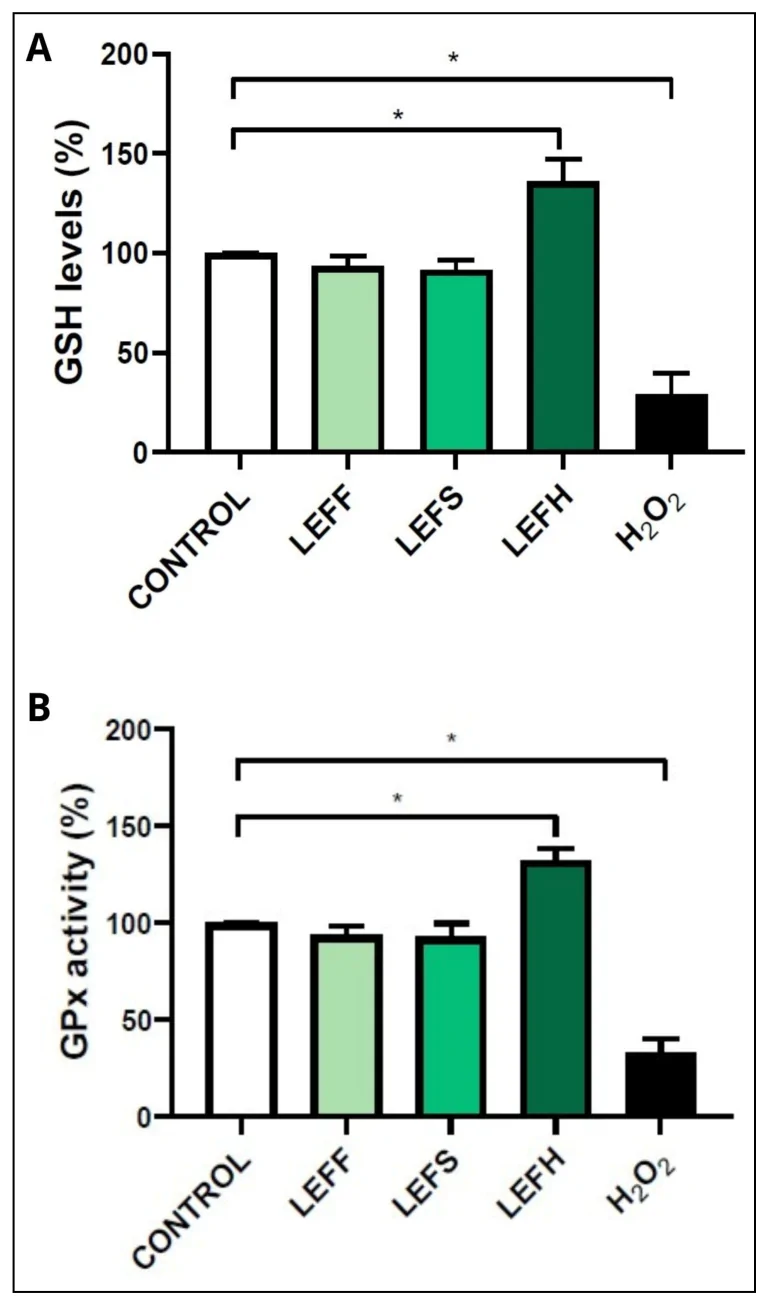
Effects of the lipid extracts on basal glutathione-dependent antioxidant parameters in hippocampal neurons. (**A**) Intracellular reduced glutathione content and (**B**) GPx activity in neurons treated with LEFF, LEFS, or LEFH. H_2_O_2_ (50 µM, 1 h) was included as an oxidative-stress control. LEFH was not evaluated in combination with H_2_O_2_ or Aβ_1–42_. Therefore, these experiments demonstrate changes in basal GSH content and GPx activity but do not establish protection against Aβ- or H_2_O_2_-induced oxidative stress. GSH and GPx results were normalized to protein content determined in paired lysate aliquots. Data are presented as mean ± SD. For GSH, six independent neuronal culture preparations were analyzed per group. For GPx activity, five independent preparations were analyzed for the control, LEFF, LEFS, and LEFH groups, and four independent preparations were analyzed for the H_2_O_2_ group. Differences among groups were assessed by one-way ANOVA followed by Bonferroni-adjusted multiple comparisons; * *p* < 0.05. All lipid extracts were applied at 0.1 µg/mL of total lipid. CONTROL denotes vehicle-treated cultures receiving 0.03% (*v*/*v*) DMSO in PBS.

Because the PPP contributes to the maintenance of cellular reducing capacity, we next examined intracellular Glutathione (GSH) content and Glutathione peroxidase (GPx) activity. LEFF and LEFS did not significantly affect either parameter relative to control neurons. In contrast, LEFH increased intracellular GSH to approximately 135% of control values (Figure 4A). A comparable response was observed for GPx, whose activity increased to approximately 130% of control values following LEFH treatment (Figure 4B). As expected, exposure to H_2_O_2_ markedly reduced both GSH content and GPx activity, confirming the responsiveness of these assays to oxidative stress.

These results show that LEFH increases basal GSH content and GPx activity. Because LEFH was not evaluated together with Aβ_1–42_ or H_2_O_2_, these findings do not demonstrate protection against induced oxidative stress.

### 2.5. LEFH Modulates AMPKα Activation, Cellular Energy Status, and Ppargc1a Expression

**Figure 5:**
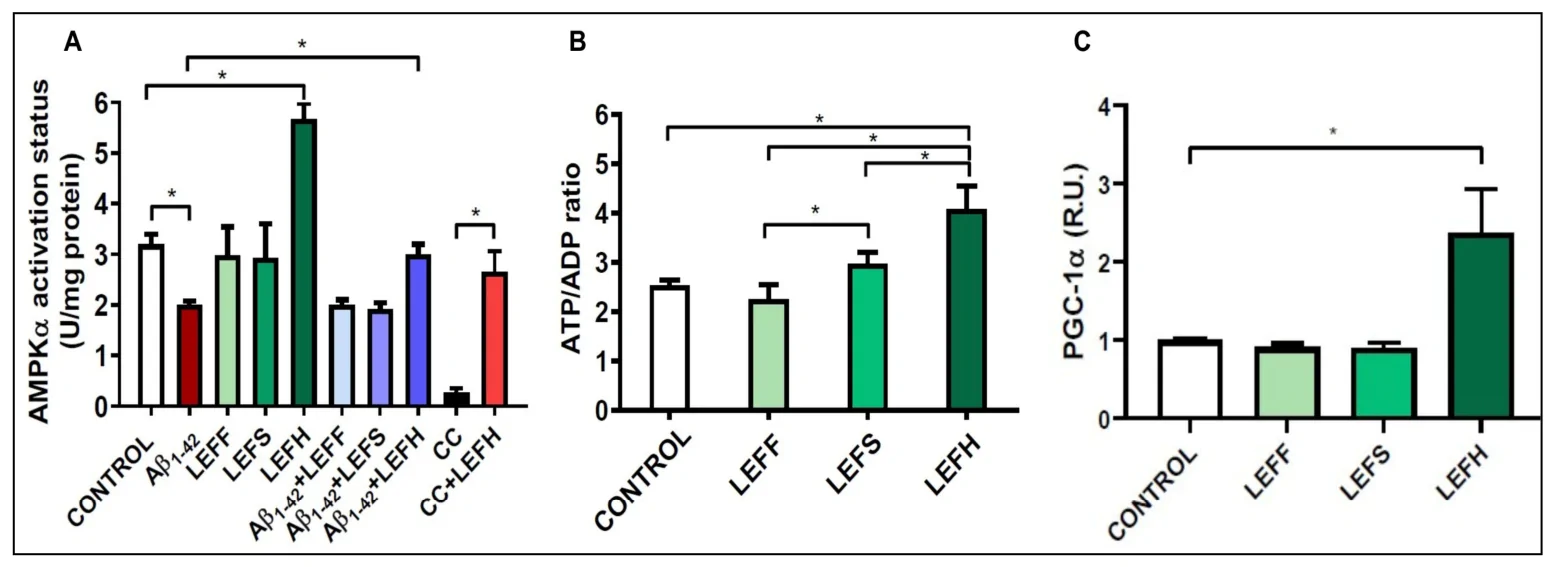
LEFH modulates AMPKα activation status, cellular energy balance, and PGC-1α expression. (**A**) AMPKα activation status assessed by quantifying AMPKα phosphorylation at Thr172 in primary hippocampal neurons treated with LEFF, LEFS, or LEFH in the absence or presence of Aβ_1–42_ preparation. Compound **C** (CC, 10 µM) was used to inhibit AMPK signaling and was evaluated alone or in combination with LEFH. Aβ_1–42_ and Compound **C** reduced phospho-AMPKα, whereas LEFH increased the signal under basal conditions and attenuated its reduction in Aβ_1–42_- or Compound **C**-treated neurons. (**B**) Cellular energy status determined from the ATP/ADP ratio in neurons treated with the three lipid extracts. (**C**) Relative Ppargc1a mRNA expression following LEFF, LEFS, or LEFH treatment. Transcript levels were normalized to cyclophilin A and expressed relative to the untreated control group. Data are presented as mean ± SD from five independent neuronal culture preparations. Technical replicates, where applicable, were averaged before statistical analysis so that each independent culture contributed a single value. In panel (**A**), the lipid extract/Aβ experimental series was analyzed by one-way ANOVA across the corresponding experimental treatment groups, whereas the Compound **C** conditions were analyzed separately by one-way ANOVA. Panels (**B**,**C**) were also analyzed by one-way ANOVA. Significant omnibus effects were followed by Bonferroni-adjusted multiple comparisons. Horizontal brackets indicate the groups compared; * *p* < 0.05. All lipid extracts were applied at 0.1 µg/mL of total lipid. CONTROL denotes vehicle-treated cultures receiving 0.03% (*v*/*v*) DMSO in PBS.

Given the effects of LEFH on neuronal glucose metabolism, we evaluated the phosphorylation of AMPKα at Thr172 as an indicator of its activation status. Aβ_1–42_ significantly reduced phospho-AMPKα relative to the control condition. LEFF and LEFS alone did not significantly modify the signal, whereas LEFH produced a marked increase above basal values. In Aβ-exposed neurons, LEFH restored phospho-AMPKα to a level close to that observed in untreated cultures (Figure 5A). Compound **C** strongly suppressed phospho-AMPKα. Notably, the addition of LEFH increased the signal relative to Compound **C** alone, indicating that LEFH retained the capacity to stimulate an AMPK-associated response under pharmacological inhibition. This experiment supports an association between LEFH and AMPKα activation but does not, by itself, demonstrate that the metabolic effects of LEFH are entirely AMPK-dependent.

Cellular energy status was evaluated by calculating the ATP/ADP ratio. LEFF produced values comparable to those of control cultures, whereas LEFS induced a modest increase. LEFH generated the strongest response, significantly elevating the ATP/ADP ratio relative to the control, LEFF, and LEFS conditions (Figure 5B). Thus, the holdfast extract increased cellular energy availability under basal conditions. Consistent with this metabolic profile, LEFH also increased PGC-1α mRNA expression by more than twofold relative to the control group. Neither LEFF nor LEFS significantly altered PGC-1α transcript levels (Figure 5C). Since PGC-1α encodes PGC-1α, this transcriptional response is compatible with the engagement of pathways involved in energy regulation and mitochondrial adaptation. However, the increase in mRNA alone does not demonstrate higher PGC-1α protein abundance or mitochondrial biogenesis.

### 2.6. LEFH Differentially Regulates Inflammatory and Metabolic Gene Expression

**Figure 6:**
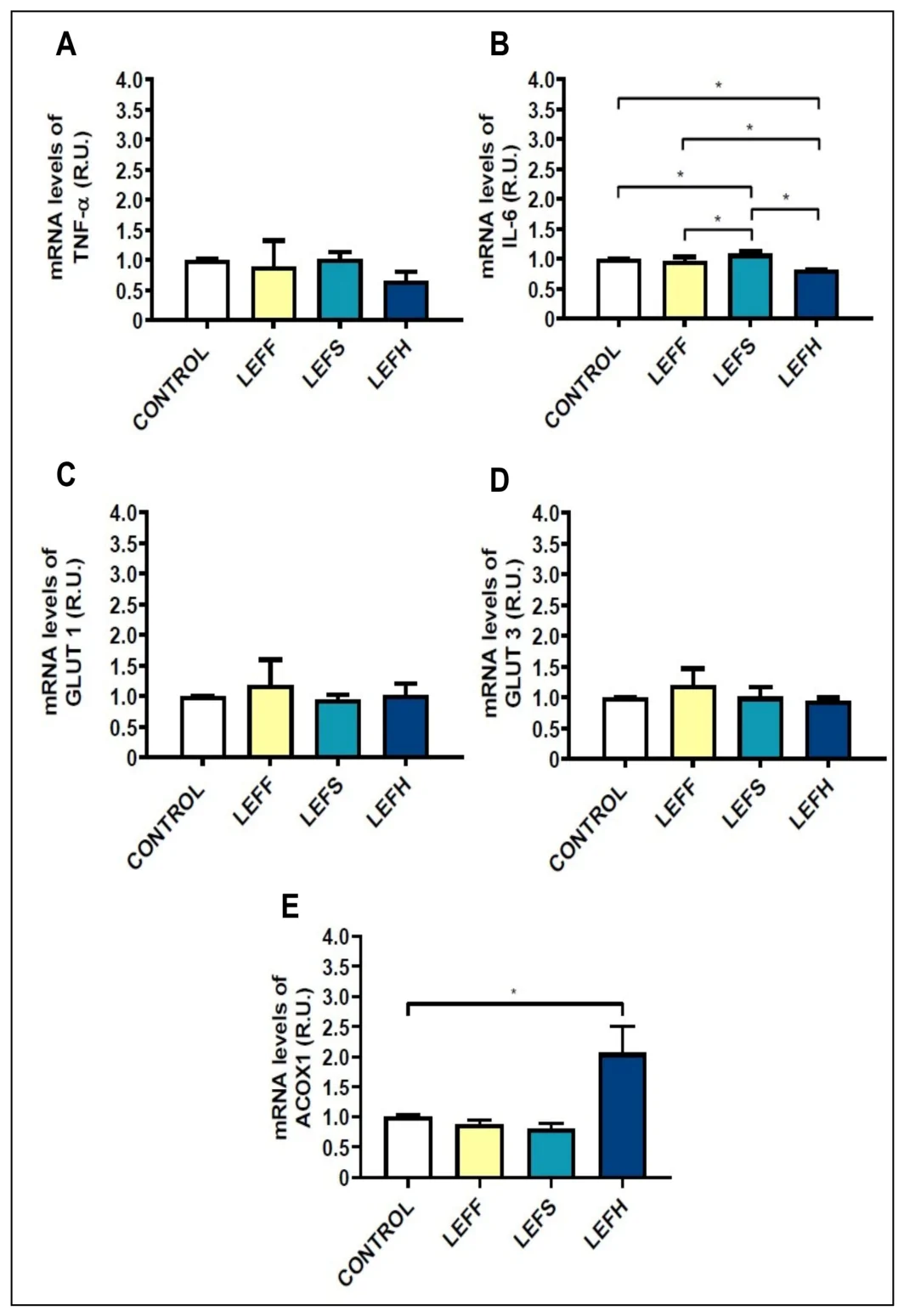
LEFH differentially regulates inflammatory and metabolic gene expression in hippocampal neurons. Relative mRNA expression of (**A**) *Tnf*-α, (**B**) IL-6, (**C**) *GLUT1*, (**D**) *GLUT3*, and (**E**) *ACOX1* in primary hippocampal neurons treated with LEFF, LEFS, or LEFH. Transcript levels were determined by quantitative real-time PCR, normalized to cyclophilin A expression, and expressed relative to the untreated control group. LEFS produced a smaller reduction in Il6 mRNA, whereas LEFH produced the largest decrease and increased *Acox1* expression without significantly modifying *Tnf*-α, *Slc2a1*, or *Slc2a3* expression. Data are presented as mean ± SD from four to five independent neuronal culture preparations, depending on the target and experimental group. Technical replicates were averaged before statistical analysis so that each independent culture contributed a single value. Differences among groups were assessed by one-way ANOVA followed by Bonferroni’s multiple-comparisons test. Horizontal brackets indicate the groups compared; * *p* < 0.05. All lipid extracts were applied at 0.1 µg/mL of total lipid. Control denotes vehicle-treated cultures receiving 0.03% (*v*/*v*) DMSO in PBS.

The transcriptional effects of the lipid extracts were examined by quantitative RT-PCR. None of the treatments significantly modified Tnf mRNA expression (Figure 6A). A different pattern was observed for Il6. LEFS produced a smaller reduction in Il6 mRNA relative to the control condition (Bonferroni-adjusted *p* = 0.030). LEFH produced the largest decrease and differed significantly from the control, LEFF, and LEFS conditions (Figure 6B). Thus, LEFH elicited the strongest effect on Il6 expression, although the response was not exclusive to this extract. Expression of *Slc2a1* and *Slc2a3*, encoding the constitutive neuronal glucose transporters GLUT1 and GLUT3, respectively, remained unchanged following treatment with any of the extracts (Figure 6C,D). Thus, the increased glucose uptake induced by LEFH was not accompanied by transcriptional upregulation of these transporters.

By contrast, LEFH increased Acox1 mRNA to approximately twice the control level, whereas LEFF and LEFS had no significant effect (Figure 6E). This finding indicates that LEFH promotes a transcriptional response involving peroxisomal lipid metabolism. Nevertheless, the increased transcript abundance does not establish higher ACOX1 enzymatic activity or increased peroxisomal β-oxidation.

### 2.7. LEFH Induces the Expression of GLUT4 and Wnt-Associated Genes

**Figure 7:**
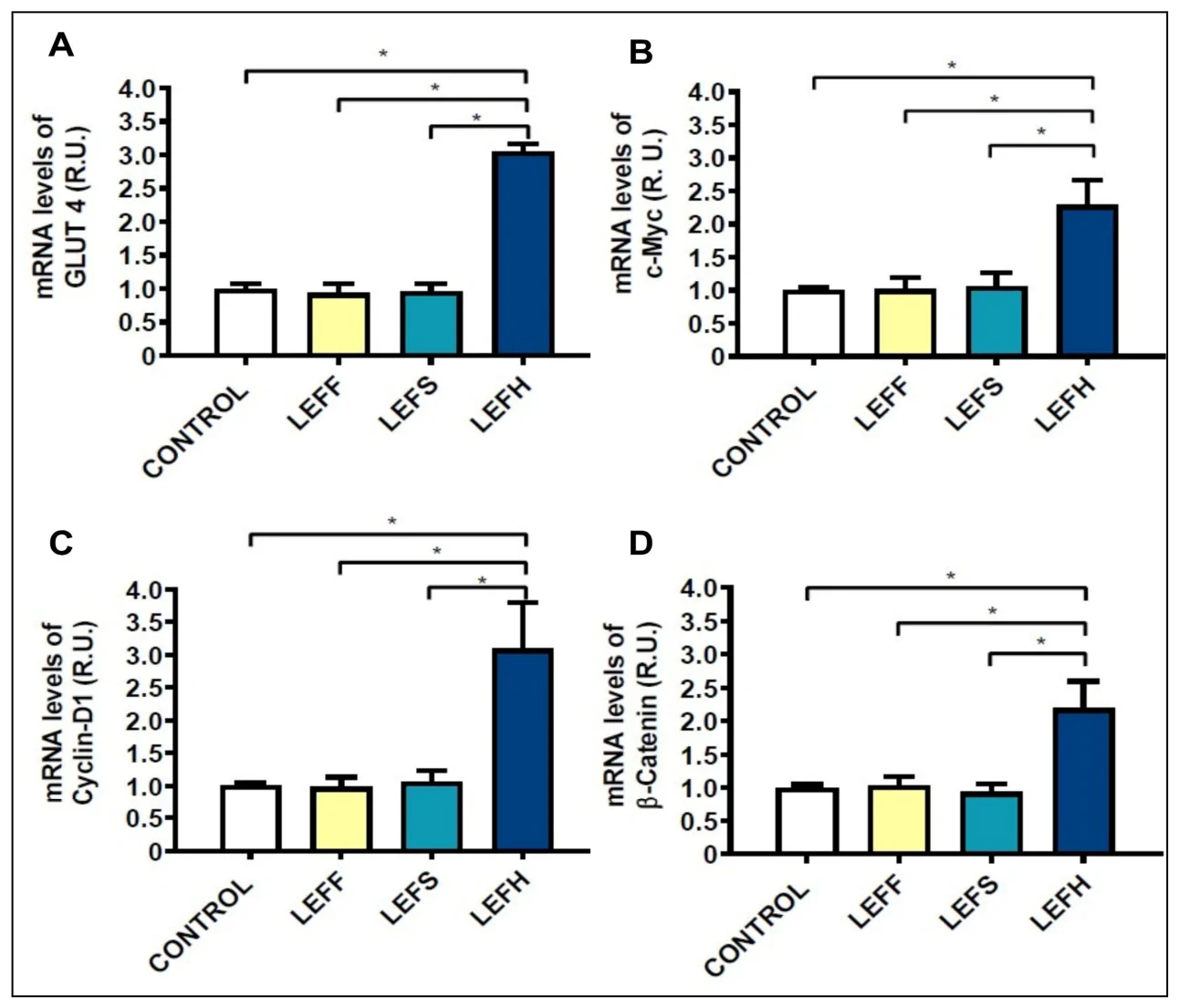
LEFH induces the expression of GLUT4 and Wnt-associated genes in hippocampal neurons. Relative mRNA expression of (**A**) Slc2a4, (**B**) *Myc*, (**C**) Ccnd1 and (**D**) β-catenin in primary hippocampal neurons treated with LEFF, LEFS, or LEFH. Transcript levels were determined by quantitative real-time PCR, normalized to cyclophilin A expression, and expressed relative to the untreated control group. LEFH significantly increased the expression of GLUT4, *Myc*, Cyclin D1, and β-catenin. Data are presented as mean ± SD from five independent neuronal culture preparations, with each condition analyzed in technical triplicate. Technical replicates were averaged before statistical analysis. Differences among groups were assessed by one-way ANOVA followed by Bonferroni’s multiple-comparisons test. Horizontal brackets indicate the groups compared; * *p* < 0.05. All lipid extracts were applied at 0.1 µg/mL of total lipid. CONTROL denotes vehicle-treated cultures receiving 0.03% (*v*/*v*) DMSO in PBS.

We next evaluated transcripts associated with glucose transport and neuronal signaling. LEFH increased GLUT4 mRNA approximately threefold relative to the control, LEFF, and LEFS groups (Figure 7A). The other extracts did not significantly affect GLUT4 expression. Since the experiment assessed mRNA, this result demonstrates transcriptional upregulation of GLUT4 but does not establish changes in GLUT4 protein abundance or membrane translocation.

LEFH also increased the expression of *Myc* and *Ccnd1*, encoding c-Myc and Cyclin D1, respectively (Figure 7B,C). In parallel, *Ctnnb1* mRNA, which encodes β-catenin, was significantly elevated following LEFH treatment (Figure 7D). LEFF and LEFS did not produce comparable responses.

The coordinated increase in *Ctnnb1*, *Myc*, and *Ccnd1* transcript levels is consistent with a Wnt-associated transcriptional profile. However, because β-catenin stabilization, nuclear localization, GSK3β regulation, and Wnt reporter activity were not evaluated, these findings should not be presented as direct evidence of canonical Wnt pathway activation.

### 2.8. LEFH Preserves Glucose Uptake and Cellular Energy Status in Hippocampal Slices

**Figure 8:**
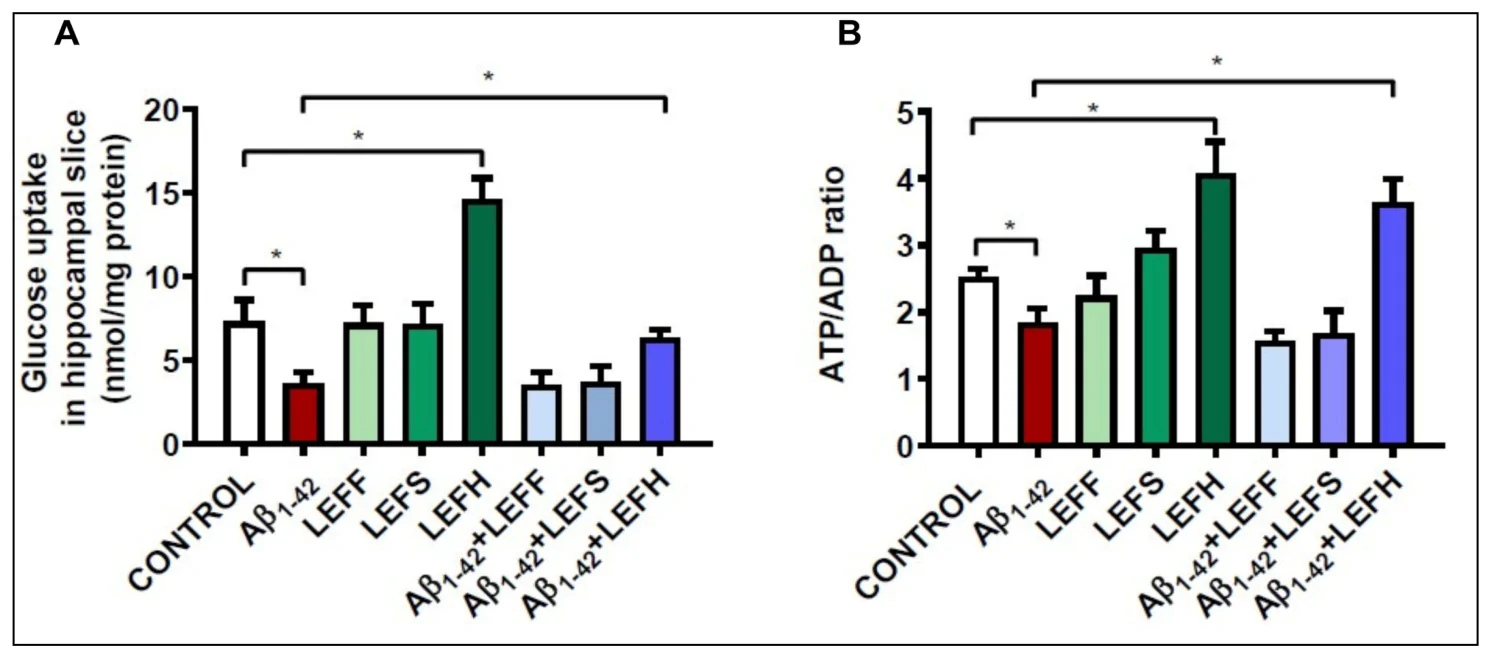
LEFH preserves glucose uptake and cellular energy status in Aβ_1–42_-challenged hippocampal slices. Acute hippocampal slices obtained from wild-type mice were treated with LEFF, LEFS, or LEFH in the absence or presence of Aβ_1–42_ preparation. (**A**) Glucose uptake measured using radiolabeled glucose and normalized to total protein content. (**B**) Cellular energy status determined from the ATP/ADP ratio. Aβ_1–42_ reduced glucose uptake and the ATP/ADP ratio, whereas LEFH increased both parameters under basal conditions and attenuated their reduction following Aβ_1–42_ exposure. Data are presented as mean ± SD from five independent hippocampal-slice preparations. Values obtained from technical replicates were averaged so that each independent tissue preparation contributed a single value to the statistical analysis. Differences among the experimental treatment groups were assessed by one-way ANOVA followed by Bonferroni-adjusted multiple comparisons. Horizontal brackets indicate the groups compared; * *p* < 0.05. All lipid extracts were applied at 0.1 µg/mL of total lipid. CONTROL denotes vehicle-treated cultures receiving 0.03% (*v*/*v*) DMSO in PBS.

The metabolic effects of the extracts were subsequently examined in ex vivo hippocampal slices. Aβ_1–42_ reduced glucose uptake by approximately 50% compared with untreated slices. LEFF and LEFS did not significantly modify basal uptake and failed to prevent the Aβ-induced decline. LEFH alone markedly increased glucose uptake, while pretreatment with LEFH significantly attenuated the reduction caused by Aβ_1–42_ and maintained uptake near control values (Figure 8A).

Aβ_1–42_ also decreased the ATP/ADP ratio, indicating impaired tissue energy status. LEFF produced no significant effect, whereas LEFS caused a moderate increase under basal conditions. LEFH generated the highest ATP/ADP ratio and significantly exceeded the control and the other extracts. In slices exposed to Aβ_1–42_, LEFH restored the ATP/ADP ratio and increased it above the value observed in the untreated control group (Figure 8B). These ex vivo findings reproduce the central metabolic effects observed in isolated neurons and show that LEFH preserves glucose handling and cellular energy balance in a system containing the cellular and structural complexity of hippocampal tissue.

## 3. Discussion

At the selected concentration of 0.1 µg/mL, the LEFH of *Macrocystis pyrifera* produced the largest biological effects among the three anatomical preparations evaluated. In primary hippocampal neurons, LEFH attenuated Aβ_1–42_-associated loss of viability and maintained glucose uptake, glycolytic flux, pentose phosphate pathway activity, and cellular energy status relative to the Aβ_1–42_-only condition. Its principal metabolic effects were reproduced in ex vivo hippocampal slices. In separate experiments performed under basal conditions, LEFH increased GSH content and GPx activity. LEFH also selectively reduced Il6 mRNA, whereas Tnf mRNA remained unchanged, and induced transcriptional changes associated with glucose and lipid metabolism. These results identify LEFH as the preparation producing the largest effects at the tested concentration but do not establish greater potency, antioxidant protection, or broad anti-inflammatory activity.

Aβ-induced disruption of neuronal energy metabolism was evident across several complementary endpoints. Exposure to Aβ_1–42_ reduced glucose uptake, glycolysis, PPP-dependent glucose oxidation, phospho-AMPKα levels, and the ATP/ADP ratio. This coordinated decline is consistent with the metabolic vulnerability described in AD, where cerebral glucose hypometabolism can arise before overt clinical manifestations and becomes more pronounced with disease progression [19,20,41]. Reduced glucose availability and utilization limit ATP production, weaken redox defenses, and compromise synaptic maintenance, thereby increasing neuronal susceptibility to amyloidogenic stress [15,42]. The ability of LEFH to preserve several interconnected metabolic pathways therefore provides a plausible basis for the protection observed in the viability assay.

The glucose uptake experiments offer important insight into the metabolic action of LEFH. The extract increased glucose uptake under basal conditions and attenuated the reduction induced by Aβ_1–42_. In the kinetic analysis, LEFH displayed the highest apparent Vmax among the groups, and the overall comparison was statistically significant. However, the Bonferroni-adjusted comparison between LEFH and control did not reach statistical significance, whereas LEFH differed significantly from LEFS. The apparent Km did not differ significantly among groups. These findings therefore suggest that LEFH may influence maximal glucose transport capacity without providing evidence for altered apparent glucose affinity. Such a response could reflect changes in transporter availability, transporter turnover, or membrane properties, although these possibilities were not directly examined in the present study.

At the transcriptional level, LEFH increased GLUT4 mRNA, whereas GLUT1 and GLUT3 remained unchanged. GLUT4 is expressed in metabolically responsive brain regions and has been associated with activity-dependent glucose utilization and neuronal function [43,44,45]. The increase in GLUT4 expression may therefore contribute to the metabolic phenotype induced by LEFH. Nevertheless, the present data do not establish that GLUT4 mediates the increase in glucose uptake, as protein abundance and membrane translocation were not assessed. The unchanged expression of GLUT1 and GLUT3 also suggests that the response may involve selective transcriptional regulation or post-transcriptional modulation of transporter activity. Future studies should examine transporter protein levels, subcellular localization, and the consequences of pharmacological or genetic inhibition of GLUT4.

LEFH also modulated AMPKα activation status. Aβ_1–42_ reduced AMPKα phosphorylation at Thr172, whereas LEFH restored it toward basal levels. In addition, Compound **C** markedly suppressed phospho-AMPKα, and the presence of LEFH increased the signal relative to Compound **C** alone. These findings support an association between LEFH exposure and AMPK-related signaling. However, they do not yet demonstrate that AMPK is required for the metabolic or neuroprotective effects of the extract. Establishing causal dependency would require showing that AMPK inhibition prevents the LEFH-induced improvement in glucose uptake, glycolysis, ATP/ADP ratio, or neuronal viability. Accordingly, the present results are more appropriately interpreted as evidence that LEFH engages an AMPK-associated metabolic response rather than acting through a fully demonstrated AMPK-dependent mechanism.

The increase in PGC-1α mRNA provides an additional link between LEFH and cellular energy regulation. PGC-1α coordinates transcriptional programs involved in mitochondrial adaptation, oxidative metabolism, and cellular responses to energetic stress. Its upregulation is compatible with a broader metabolic response downstream or parallel to AMPK signaling. Nevertheless, because only transcript levels were measured, the present findings do not establish an increase in PGC-1α protein, transcriptional activity, mitochondrial content, or respiratory capacity. Similarly, the higher ATP/ADP ratio observed after LEFH treatment reflects improved cellular energy status but should not be interpreted as direct evidence of increased mitochondrial ATP synthesis. Measurements of oxygen consumption, mitochondrial membrane potential, respiratory-chain activity, and PGC-1α protein would help define whether mitochondrial remodeling contributes to the response.

Preservation of PPP activity may support glutathione recycling through NADPH production. In separate experiments performed under basal conditions, LEFH increased GSH content and GPx activity. However, LEFH was not evaluated together with Aβ_1–42_ or H_2_O_2_, NADPH and ROS were not measured directly, and residual BHT was not quantified. Therefore, these findings demonstrate an increase in basal glutathione-associated parameters but do not establish protection against Aβ- or H_2_O_2_-induced oxidative stress. Future studies should directly assess cytosolic and mitochondrial ROS, lipid peroxidation, oxidative damage, and glutathione redox status in Aβ-treated neurons using an appropriate BHT-matched control. The transcriptional response induced by LEFH extended beyond glucose metabolism. The extract increased Ctnnb1, *Myc*, and Ccnd1 mRNA expression. These genes are associated with canonical Wnt/β-catenin signaling, a pathway involved in neuronal survival, synaptic maintenance, and metabolic regulation [46]. Their coordinated upregulation is therefore compatible with a Wnt-associated transcriptional response. However, the evidence is not sufficient to conclude that LEFH activates canonical Wnt signaling. β-Catenin stabilization, nuclear accumulation, Glycogen synthase kinase 3 beta (GSK3β) regulation, transcriptional reporter activity, and sensitivity to Wnt inhibitors were not evaluated. The changes observed here should consequently be considered hypothesis-generating and provide a rationale for more direct pathway validation.

LEFH also reduced *Il6* mRNA expression without significantly altering *Tnf*. This selective response suggests that the extract modulates specific inflammatory-associated transcriptional responses rather than producing broad suppression of inflammatory signaling. IL-6 participates in both physiological and pathological responses in the nervous system, and sustained elevation has been associated with neuroinflammation and metabolic dysfunction in AD [47,48]. Nevertheless, because the experiments were performed in neuron-enriched cultures and did not include microglial or astrocytic inflammatory responses, the reduction in *Il6* should not be interpreted as direct evidence that LEFH suppresses neuroinflammation in vivo. A more complete evaluation will require mixed cultures or animal models that preserve interactions among neurons, astrocytes, microglia, and vascular cells.

The increase in ACOX1 mRNA indicates that the effects of LEFH may also involve peroxisomal lipid metabolism. ACOX1 catalyzes the first step of peroxisomal β-oxidation and contributes to the processing of very-long-chain fatty acids and cellular lipid homeostasis. Its transcriptional upregulation, together with the increase in PGC-1α, suggests a coordinated adaptation involving energy and lipid metabolic pathways [49]. However, increased Acox1 expression does not necessarily imply higher enzymatic activity or greater peroxisomal β-oxidation. Measurements of ACOX1 protein, peroxisomal function, and fatty acid oxidation will be required to determine the functional importance of this response.

The differential activity of the three extracts may be related to their lipid composition. Fronds, stipes, and holdfasts showed distinct proportions of saturated, monounsaturated, and polyunsaturated fatty acids, and LEFH was particularly enriched in monounsaturated fatty acids relative to the frond-derived extract. Nevertheless, the biological activity observed in the present study cannot be attributed to a single lipid or lipid class based on the current chemical analysis. LEFH should therefore be considered a chemically complex bioactive preparation rather than a single-compound intervention. Importantly, the biological activity of complex natural-product extracts does not necessarily correspond to the activity of one predominant constituent. Additive, synergistic, and antagonistic interactions among multiple components can modify the overall pharmacological response of natural-product mixtures [50,51,52]. This consideration is particularly relevant to marine extracts. In edible seaweed preparations, fractionation has been reported to reduce antioxidant activity relative to the corresponding unfractionated extracts, suggesting that interactions among multiple constituents may contribute to the activity of the intact preparation [52,53]. Therefore, purification of a complex marine extract does not necessarily preserve the biological phenotype of the original preparation.

Accordingly, the stronger activity of LEFH compared with LEFF and LEFS may reflect differences in its overall chemical composition and interactions among multiple constituents rather than the presence of a single dominant active molecule. However, synergistic interactions have not been directly demonstrated for LEFH and should not be inferred from the present results. Establishing this possibility will require comprehensive chemical profiling followed by bioactivity-guided fractionation, testing of purified candidate components, and, ideally, reconstitution experiments comparing the intact extract with isolated and recombined constituents. These approaches will be necessary both to identify the chemical determinants of LEFH activity and to determine whether its biological effects depend on individual molecules, additive interactions, or true synergistic relationships.

The chemical complexity of LEFH also has important implications for structure–activity relationship and pharmacological interpretation. At the present stage, a conventional structure–activity relationship analysis and a meaningful assessment of drug-likeness cannot yet be performed because the constituent or combination of constituents responsible for the observed biological phenotype has not been established. As the chemical composition of LEFH and its relevant active and/or marker constituents become better defined, these properties can be evaluated more appropriately [53].

Pharmacokinetic characterization will also represent an important subsequent step because individual constituents of a complex natural preparation may differ in absorption, systemic distribution, metabolic stability, clearance, and tissue exposure. Once the principal LEFH constituents and bioactivity-associated fractions have been defined, subsequent studies should evaluate their physicochemical properties, metabolic stability, systemic exposure, and brain distribution. The present in vitro and ex vivo study was designed to establish biological activity and was not intended to determine bioavailability, pharmacokinetics, or blood–brain barrier penetration; these parameters will be required before the translational potential of LEFH can be established [33,54].

Available compound-level information provides only limited context for interpreting the present findings. Myristic acid (C14:0), palmitic acid (C16:0), and oleic acid (C18:1n-9c) are common dietary and endogenous fatty acids that enter general pathways of intestinal absorption, esterification, lipoprotein- or albumin-mediated transport, tissue uptake, and oxidation. More specific experimental information is available for EPA and erucic acid. EPA enters the brain but is rapidly β-oxidized, which limits its accumulation in brain phospholipids. Plasma-derived erucic acid also crosses the rat blood–brain barrier, although its cerebral uptake is limited and it undergoes rapid metabolism after entering the brain [55,56]. These compound-level observations cannot be extrapolated directly to LEFH because the extract contains a mixture of molecular lipid species and other unresolved lipophilic constituents. Accordingly, the present fatty-acid profile neither identifies the bioactive component nor predicts the absorption, bioavailability, metabolism, or brain exposure of the intact preparation.

Quality control and standardization represent additional requirements for the future development of LEFH as a reproducible bioactive preparation. In the present proof-of-concept study, analytical reproducibility was addressed at the extraction level by processing three independent samples from each anatomical structure, determining total lipid yield gravimetrically, and characterizing the fatty-acid profile by GC-FID using a 37-component FAME reference standard. These procedures allowed us to compare the principal lipid composition of independently prepared extracts within the experimental material used in this study. However, this approach should not be considered equivalent to a validated batch-to-batch standardization protocol, because the current material originated from a single collection campaign and the complete molecular lipid composition of LEFH has not yet been established.

For chemically complex natural preparations, reproducible development requires a combination of authenticated source material, standardized extraction procedures, quantitative marker analysis, and chemical fingerprinting capable of capturing the broader compositional profile [53,57]. Chromatographic fingerprinting combined with multivariate analysis is particularly useful because it evaluates patterns across multiple constituents rather than relying exclusively on a single marker [58]. For lipid-rich marine extracts, LC-MS-based lipidomic approaches provide an additional advantage by resolving and annotating multiple lipid classes that are not captured by fatty-acid methyl ester analysis alone; UHPLC-MS/MS approaches have been successfully applied to the in-depth characterization of lipid-rich brown-seaweed extracts [59]. A future LEFH standardization strategy should therefore integrate the current GC-FID fatty-acid profile with comprehensive LC-MS-based fingerprinting, selected quantitative chemical markers, internal quality-control samples, and predefined acceptance criteria for compositional similarity among independently collected and prepared batches. Importantly, such batch-to-batch validation will require prospectively generated independent harvest and extraction batches and cannot be established rigorously from a single collection campaign.

Formulation represents an additional and distinct challenge for the further development of LEFH. The use of DMSO in the present experiments was restricted to in vitro and ex vivo solubilization and should not be interpreted as a proposed pharmaceutical formulation. Because LEFH is a chemically complex lipid-rich preparation, the optimal formulation will depend on its final chemical profile, the identity and physicochemical properties of the bioactivity-associated constituents, and the intended route of administration. Relevant parameters will include aqueous dispersibility, oxidative and storage stability, compatibility with excipients, release behavior, gastrointestinal stability when oral administration is considered, systemic exposure, and preservation of biological activity after formulation.

Lipid-based and colloidal delivery systems, including nanoemulsions, liposomes or nanophytosomes, solid lipid nanoparticles, and nanostructured lipid carriers, have been investigated as approaches to improve the solubility, stability, bioavailability, and tissue delivery of poorly water-soluble neuroprotective compounds and natural-product extracts [60]. Such approaches may also be relevant to marine-derived compounds. For example, delivery systems developed for fucoxanthin, a lipophilic constituent of brown algae, have improved its stability, release, and bioaccessibility relative to the non-formulated compound [61,62]. These studies illustrate the potential value of formulation strategies for algae-derived lipophilic bioactives but cannot be directly extrapolated to LEFH, which contains a more complex and incompletely characterized mixture.

Central nervous system delivery introduces an additional pharmacological barrier because systemic exposure does not necessarily translate into effective brain exposure. The blood–brain barrier restricts the entry of many bioactive compounds, and lipid-based nanocarriers have therefore been explored to enhance brain delivery of neuroprotective drugs and extracts [60]. Before selecting a delivery platform for LEFH, comparative formulation studies should therefore evaluate physicochemical stability, encapsulation or incorporation efficiency, particle size and dispersity where applicable, release characteristics, preservation of biological activity, systemic pharmacokinetics, and brain exposure. Future chemical characterization and in vivo evaluation of the intact extract will provide an important basis for selecting an appropriate route of administration and subsequent formulation strategy.

The results obtained in hippocampal slices strengthen the biological relevance of the findings. Unlike isolated neuronal cultures, slices retain local cellular diversity, extracellular architecture, and neuronal–glial interactions. LEFH preserved glucose uptake and the ATP/ADP ratio in Aβ-treated slices, reproducing the main metabolic effects observed in cultured neurons. This consistency across experimental systems reduces the likelihood that the response is restricted to a particular culture condition. However, acute slices cannot reproduce absorption, distribution, metabolism, blood–brain barrier penetration, chronic toxicity, or behavioral outcomes. In vivo studies will therefore be essential to determine whether the active components reach the brain and preserve metabolic and cognitive function in animal models of AD.

Several limitations should be considered. A major limitation of the present study is the absence of detailed chemical identification and characterization of the constituent or combination of constituents responsible for the biological activity of LEFH. The current GC-FID analysis defines the fatty-acid profile but does not resolve intact molecular lipid species, other lipophilic constituents, or the specific bioactive components of the extract.

The study used an acute Aβ_1–42_ preparation generated under oligomer-forming conditions and did not evaluate tau phosphorylation, APP processing, synaptic markers, or chronic neurodegenerative changes. The present experimental design specifically evaluated the protective effect of lipid-extract pretreatment and therefore does not establish whether LEFH can reverse metabolic dysfunction once Aβ-associated alterations are already established. A post-insult or reversal protocol represents an important next step to distinguish preventive/protective activity from true reversal of established neuronal metabolic dysfunction. The Aβ_1–42_ preparation was generated using an established oligomerization protocol previously applied in neuronal models [63,64,65]. Nevertheless, the molecular-size distribution and oligomeric composition of the specific preparation used in the present study were not independently characterized. Therefore, the term ‘Aβ_1–42_ preparation’ denotes the operational preparation generated using this standardized protocol rather than a quantitatively defined individual oligomeric species. Moreover, GLUT4 regulation and Wnt signaling were assessed only at the transcript level; the Compound **C** experiment interrogated AMPK-associated signaling but did not establish whether AMPK inhibition abolishes the metabolic or neuroprotective effects of LEFH; and oxidative stress was inferred from antioxidant parameters rather than measured directly.

A further limitation is that the lipid extracts were evaluated at a single working concentration (0.1 µg/mL). Concentration–response analyses will be required to define the potency of LEFH, to establish whether its metabolic and neuroprotective effects are concentration-dependent, and to determine the concentration range over which the preparation remains well tolerated. The magnitude of the metabolic and transcriptional responses obtained at this low total-lipid concentration also suggests that the activity of LEFH may reside in a minor constituent, or in a limited subset of constituents present at low relative abundance, rather than in its most abundant fatty acids. This consideration provides an additional rationale for the bioactivity-guided fractionation and LC-MS-based profiling strategy outlined above.

More broadly, the signaling measurements in the present study should be interpreted as pathway-associated rather than as evidence of pathway dependency. The coordinated changes in phospho-AMPKα, *Ppargc1a*, *Slc2a4*, *Ctnnb1*, *Myc*, and *Ccnd1* identify candidate mechanisms associated with the LEFH-induced metabolic phenotype, but they do not establish a linear causal pathway. Definitive mechanistic attribution will require functional loss-of-function approaches demonstrating that disruption of the corresponding pathway prevents the metabolic or neuroprotective effects of LEFH. Thus, the present study defines a reproducible biological and metabolic phenotype and generates specific mechanistic hypotheses for subsequent causal testing. These limitations do not diminish the metabolic effects observed but define the scope of the conclusions and the experiments needed to clarify the underlying mechanisms.

Overall, the findings support LEFH as a promising marine-derived modulator of neuronal metabolism under amyloidogenic stress. Its effects encompass preservation of glucose uptake and utilization, maintenance of cellular energy balance, enhancement of glutathione-dependent defenses, and regulation of metabolic and signaling-associated genes. Rather than acting through a single established pathway, LEFH appears to induce a coordinated adaptive response involving glucose handling, energy sensing, redox regulation, and lipid metabolism. Chemical standardization, mechanistic validation, and chronic in vivo assessment are now required to determine whether this complex lipid extract, or specific molecules derived from it, can be developed as a complementary strategy for AD-related metabolic dysfunction.

## 4. Materials and Methods

### 4.1. Macroalgae Materials

*Macrocystis pyrifera* is a dominant component of sub-Antarctic kelp forests in the Magellan region of southern Chile (47–56° S, 71–73° W). This region is characterized by low temperatures, variable salinity, pronounced seasonal changes in photoperiod and solar radiation, and high benthic marine biodiversity [66,67]. Specimens were collected in November 2021 and processed immediately after collection. After separation into fronds, stipes, and holdfasts, the material was lyophilized and maintained under controlled frozen storage until lipid extraction (−120 °C). Lipid extraction and fatty-acid characterization were performed in November 2022, whereas the neuronal and hippocampal-slice experiments using the resulting lipid preparations were conducted between January 2024 and August 2024.

### 4.2. Lipid Extraction, Fatty Acid Methyl Ester Preparation, and Gas Chromatography Analysis

Total lipids were extracted from lyophilized fronds, stipes, and holdfasts using the method described by Bligh and Dyer (1959) [68]. For each anatomical structure, three independent 3.0 g samples were analyzed, resulting in a total of nine samples. The material was ground and homogenized for 3 min in a CHCl_3_:CH_3_OH:H_2_O mixture at a volume ratio of 1:2:0.8, corresponding to 10, 20, and 8 mL, respectively, and subsequently vacuum-filtered. A second extraction was performed to maximize lipid recovery. The solvent ratio was then adjusted to 2:2:1.8 to induce phase separation, and the samples were allowed to separate for 10 min. The chloroform phase containing the lipid extract was recovered and concentrated under reduced pressure using a rotary evaporator at 45 °C for 20 min. Residual solvent was removed under a nitrogen stream. Total lipid content was determined gravimetrically, and the resulting extracts were stored in a chloroform solution containing 0.01% butylated hydroxytoluene under a nitrogen atmosphere, protected from light, at −80 °C until further analysis [36]. Before biological use, extract aliquots were evaporated to dryness under reduced pressure and nitrogen and reconstituted in DMSO. Chloroform was not added to the cultures or slices, and all three extracts underwent the same solvent-exchange procedure. Residual BHT was not quantified after solvent exchange; therefore, trace carryover into the experimental preparations cannot be excluded.

These storage conditions were selected to minimize lipid oxidation and compositional changes during the interval between extraction and biological analysis. Low-temperature storage is particularly important for preserving unsaturated fatty acids in macroalgal material. In freeze-dried seaweeds, Schmid et al. demonstrated that total fatty-acid and PUFA levels remained stable during prolonged storage at −20 °C, whereas storage at higher temperatures resulted in greater compositional changes [69]. Consistent with these observations, long-term studies of several seaweed species have reported substantial stability of fatty-acid and overall chemical profiles during storage, although the magnitude of preservation may depend on species and storage conditions [70]. In addition, the inclusion of BHT during low-temperature storage provides further protection against oxidative modification of unsaturated fatty acids [71]. Accordingly, the lipid extracts used in the present study were maintained at −80 °C in the presence of BHT, under nitrogen and protected from light, and repeated freeze–thaw cycles were avoided. Nevertheless, because a dedicated longitudinal stability analysis of the LEFH preparation was not performed over the complete storage period, subtle time-dependent changes in minor lipid species cannot be completely excluded.

For fatty acid profiling, an aliquot of each dried lipid extract was converted into fatty acid methyl esters (FAMEs) according to the procedure described by Metcalfe et al. (1966) [72]. The resulting hexane phases were pooled and concentrated to a final volume of 1 mL under a nitrogen stream.

FAMEs were analyzed using an Agilent 7890B gas chromatograph equipped (Agilent Technologies, Santa Clara, CA, USA) with an autosampler and a flame ionization detector. Separation was performed on an Agilent HP-88 capillary column (60 m × 0.25 mm internal diameter × 0.20 µm film thickness). Individual FAMEs were identified by comparing their retention times with those of a high-purity 37-component FAME standard mixture (Sigma-Aldrich, St. Louis, MO, USA), as previously described [36].

### 4.3. Mouse Hippocampal Neuronal Cultures

Primary hippocampal neuronal cultures were prepared from embryonic day 17.5 (E17.5) mouse embryos. Pregnant C57BL/6J females were deeply anesthetized with isoflurane and euthanized by decapitation. The embryos were then removed, and their brains were collected under sterile conditions. Hippocampi were dissected in ice-cold phosphate-buffered saline (PBS), mechanically minced, and incubated with 1% trypsin for 10 min at 37 °C. Following enzymatic digestion, the tissue was gently dissociated by repeated pipetting, and the resulting cell suspension was centrifuged at 300× *g* for 1 min. Cells were seeded onto poly-L-lysine-coated culture plates in low-glucose Dulbecco’s Modified Eagle’s Medium (DMEM) and maintained for 30 min at 37 °C in a humidified atmosphere containing 5% CO_2_. The medium was subsequently replaced with Neurobasal medium supplemented with B27, penicillin, and streptomycin. Hippocampi obtained during each independent dissection were pooled before enzymatic and mechanical dissociation. Each culture generated from a separate tissue pool was considered one independent biological preparation. Wells derived from the same preparation were considered within-preparation replicates and were averaged before statistical analysis. Because hippocampi were pooled before culture preparation, embryos were not analyzed separately according to sex.

Cultures were maintained for 14 days in vitro (DIV14) before experimental use. Prior to treatment, neurons were washed with PBS and incubated in Neurobasal medium containing the lipid extracts or other experimental compounds at the concentrations and exposure times specified for each assay. Neuronal enrichment, exceeding 95%, was confirmed in randomly selected cultures by immunofluorescence staining for microtubule-associated protein 2 (MAP2) and glial fibrillary acidic protein (GFAP), as previously described [63,73]. The Committee for Ethics of Animal Experiments approved the experiments, which were carried out in accordance with the Guidelines for Animal Experiments, P. Universidad Católica de Chile, and the Manual of Biosafety Standards and Associated Risks, Comisión Nacional de Investigación Científica y Tecnológica (CONICYT).

### 4.4. Preparation of Hippocampal Slices

Transverse slices of the dorsal hippocampus, 350 µm in thickness, were prepared in ice-cold artificial cerebrospinal fluid containing 119 mM NaCl, 26.2 mM NaHCO_3_, 2.5 mM KCl, 1 mM NaH_2_PO_4_, 1.3 mM MgCl_2_, 10 mM glucose, and 2.5 mM CaCl_2_. After sectioning, the slices were allowed to recover for 1 h at 22 °C in the same solution before experimental treatment, as previously described [74]. Each biological replicate was obtained from a different animal, and slices assigned to different experimental conditions were not obtained from the same animal. When multiple slices from one animal were analyzed under the same condition, their measurements were averaged so that each animal contributed one biological value.

### 4.5. Preparation of Aβ_1–42_ Under Oligomer-Forming Conditions

Synthetic Aβ_1–42_ peptide corresponding to the wild-type human sequence was obtained from Genemed Synthesis, Inc. (custom synthesis, lot no. 105053, San Antonio, TX, USA). Lyophilized peptide was initially dissolved in 1,1,1,3,3,3-hexafluoro-2-propanol (HFIP; cat. no. H-8508, Sigma-Aldrich, St. Louis, MO, USA) and incubated for 1 h at 22 °C to disrupt pre-existing aggregates. The solvent was then removed by lyophilization, and the resulting peptide film was stored until oligomer preparation. To prepare Aβ_1–42_ under oligomer-forming conditions, the peptide film was dissolved in dimethyl sulfoxide (DMSO; cat. no. W387520, Sigma-Aldrich) and subsequently diluted in distilled water to a final peptide concentration of 100 µM, following previously described procedures [64].

The oligomerization procedure followed the established protocol described by Stine et al. [64] and previously applied in neuronal studies [65,75]. Because the molecular-size distribution and oligomeric composition of the specific preparation used in this study were not independently characterized, it is referred to as an ‘Aβ_1–42_ preparation generated under oligomer-forming conditions’ rather than as an analytically confirmed individual oligomeric species.

### 4.6. Experimental Treatments

Primary hippocampal neurons and hippocampal slices were pretreated with the corresponding lipid extract for 1 h at a final concentration of 0.1 µg/mL. The 0.1 µg/mL concentration was selected from a preliminary concentration-range viability assessment because it did not produce detectable cytotoxicity. This assessment was performed for dose selection and not as an efficacy dose–response experiment. Each dried lipid extract was prepared as a stock solution containing 10 µg/mL of total lipid and 3% DMSO (*v*/*v*). Immediately before treatment, the stock was diluted 1:100 in the experimental medium, resulting in a final lipid-extract concentration of 0.1 µg/mL and a final DMSO concentration of 0.03% (*v*/*v*). Vehicle-control samples received the same final DMSO concentration. Extract concentrations were normalized to the gravimetrically determined total lipid content of each preparation so that LEFF, LEFS, and LEFH were compared at equivalent total-lipid mass. DMSO was used exclusively as an experimental vehicle and should not be interpreted as a proposed pharmaceutical formulation.

After the 1 h pretreatment period, the Aβ_1–42_ preparation was added without removing the lipid extract, and both treatments remained present for an additional 5 h. Therefore, the total exposure to the lipid extract was 6 h. Samples assigned to the Aβ-only condition received 5 µM Aβ_1–42_ for the same period without prior exposure to the lipid extracts. Control samples received the corresponding vehicle under equivalent experimental conditions [63,76]. The treatment intervals were selected to model an acute protective paradigm. The 1 h pretreatment period was chosen to allow sufficient exposure of neurons and hippocampal tissue to the lipid extracts before the amyloidogenic challenge, while avoiding prolonged pre-exposure. A 1 h treatment interval has previously been shown to be sufficient to induce measurable changes in glucose uptake, glycolysis, ATP production, and AMPK-related signaling in primary hippocampal neurons and hippocampal slices [63]. The subsequent 5 h exposure to 5 µM Aβ_1–42_ was selected as an acute amyloidogenic-stress paradigm. This same concentration and exposure time has been used in primary hippocampal neurons to induce Aβ oligomer-associated synaptic alterations and in cultured hippocampal and cortical neurons to induce Aβ_1–42_-dependent neuronal dysfunction [65]. In addition, short-term exposure to Aβ oligomers has been shown to disrupt neuronal AMPK signaling and energy metabolism within a 3–12 h experimental window [75]. Thus, the selected conditions were intended to capture acute Aβ-associated metabolic dysfunction while permitting evaluation of the protective effects of the lipid extracts.

### 4.7. Assessment of Neuronal Viability by Nuclear Staining

Following treatment, neuronal cultures were washed with PBS and fixed with 4% paraformaldehyde (cat. no. 1040051000, Merck, Kenilworth, NJ, USA). Cells were then incubated with Hoechst dye (cat. no. H3570, Thermo Fisher Scientific, Waltham, MA, USA) for 30 min at 22 °C, washed with PBS, and examined by fluorescence microscopy. Neuronal viability was determined by evaluating nuclear morphology. Cells exhibiting condensed or fragmented nuclei were classified as non-viable, whereas cells with intact and uniformly stained nuclei were considered viable, as previously described [77].

### 4.8. Glucose Uptake Assay

Glucose uptake was measured in primary hippocampal neuronal cultures and hippocampal slices using radiolabeled 2-deoxy-D-glucose (2-DG). Following the corresponding treatments, samples were washed twice with uptake buffer containing 135 mM NaCl, 15 mM HEPES, 5 mM KCl, 1.8 mM CaCl_2_, and 0.8 mM MgCl_2_, adjusted to pH 7.4. Uptake was initiated by adding 1–1.2 µCi of 2-deoxy-D-[1,2-^3^H(N)]-glucose ([^3^H]-2-DG; PerkinElmer, MA, USA).

For time-course experiments, samples were incubated for the indicated periods up to 180 s. Uptake was terminated by rapidly adding ice-cold stop buffer consisting of uptake buffer supplemented with 0.2 mM HgCl_2_. Neuronal cultures were lysed in 1 mL of 10 mM Tris-HCl containing 0.2% SDS, pH 8.0. Hippocampal slices were homogenized in the same lysis buffer. Three milliliters of scintillation fluid were added to each sample, and incorporated radioactivity was quantified using a Tri-Carb 2900TR liquid scintillation analyzer (PerkinElmer, Shelton, CT, USA).

Glucose uptake in the neuronal time-course and Aβ-challenge experiments was normalized to total protein content and expressed as nmol/mg protein. In the neuronal concentration-response experiments used to estimate the apparent kinetic parameters, glucose uptake was normalized to cell number and expressed as pmol/10^6^ cells/min. Uptake in hippocampal slices was normalized to total protein content.

### 4.9. Determination of Glycolytic Flux

Glycolytic flux was quantified by measuring the production of ^3^H_2_O from [3-^3^H]-glucose, as previously described [78]. Following treatment, neuronal cultures or hippocampal slices were washed twice with Krebs–Henseleit buffer containing 11 mM Na_2_HPO_4_, 122 mM NaCl, 3.1 mM KCl, 0.4 mM KH_2_PO_4_, 1.2 mM MgSO_4_, and 1.3 mM CaCl_2_, adjusted to pH 7.4.

Samples were equilibrated for 30 min at 37 °C in 0.5 mL of Hank’s balanced salt solution containing 5 mM glucose. An additional 0.5 mL of the same solution containing [3-^3^H]-glucose (cat. no. NET331C250UC; PerkinElmer, MA, USA) was then added to achieve a final specific activity of approximately 1–3 disintegrations/min/pmol. After incubation, 100 µL aliquots were transferred to open microcentrifuge tubes placed inside sealed scintillation vials containing 0.5 mL of distilled water. The vials were incubated at 45 °C for 48 h to allow vapor-phase equilibration of the generated ^3^H_2_O. The inner tubes were subsequently removed, scintillation fluid was added, and radioactivity was measured for 5 min using a liquid scintillation counter. Glycolytic flux was normalized to total protein content.

### 4.10. Quantification of ATP and ADP

ATP levels were measured in neuronal cultures and hippocampal slices using an ATP determination kit (cat. no. A22066; Invitrogen/Molecular Probes, Waltham, MA, USA), according to the manufacturer’s instructions. ADP was quantified using a commercial ADP assay kit (cat. no. ab83359; Abcam, Cambridge, UK). ATP and ADP were measured in samples collected immediately after the corresponding treatments. The ATP/ADP ratio was calculated for each biological replicate and used as an indicator of cellular energy status, as previously described [79].

### 4.11. Measurement of Glucose Oxidation Through the Pentose Phosphate Pathway (PPP)

Glucose oxidation through the PPP was determined from the differential production of ^14^CO_2_ from D-[1-^14^C]-glucose and D-[6-^14^C]-glucose, as previously described [78]. Carbon 1 of glucose is released as CO_2_ during both oxidative PPP metabolism and mitochondrial oxidation, whereas carbon 6 is released predominantly during mitochondrial oxidation. PPP-dependent glucose oxidation was therefore calculated by subtracting the ^14^CO_2_ generated from D-[6-^14^C]-glucose from that generated from D-[1-^14^C]-glucose. Following treatment, neuronal cultures or hippocampal slices were washed with ice-cold PBS and transferred to Erlenmeyer flasks containing oxygen-saturated Krebs–Henseleit buffer composed of 11 mM Na_2_HPO_4_, 122 mM NaCl, 3.1 mM KCl, 0.4 mM KH_2_PO_4_, 1.2 mM MgSO_4_, and 1.3 mM CaCl_2_, pH 7.4. Samples were incubated in 0.5 mL of buffer containing 5.5 mM D-glucose and either 0.5 µCi of D-[1-^14^C]-glucose or 2 µCi of D-[6-^14^C]-glucose. Each flask contained a central well holding a microcentrifuge tube with 500 µL of benzethonium hydroxide to trap the released ^14^CO_2_. Flasks were flushed with oxygen for 20 s, sealed, and incubated for 60 min at 37 °C with continuous shaking. Reactions were terminated by injecting 0.2 mL of 1.75 M perchloric acid into the main compartment. Shaking was continued for an additional 20 min to ensure complete trapping of ^14^CO_2_. Radioactivity retained in the benzethonium hydroxide was quantified by liquid scintillation counting.

### 4.12. Measurement of Intracellular GSH and GPx Activity

Following treatment, neuronal cultures were washed with PBS and lysed with 1% sulfosalicylic acid. A paired lysate aliquot collected before deproteinization was used to determine protein content, and the corresponding GSH value was normalized to this measurement. Lysates were centrifuged at 13,000× *g* for 5 min at 4 °C, and the resulting supernatants were used for GSH quantification. Total GSH content was measured in 96-well plates using a reaction mixture containing 0.1 M phosphate buffer, 0.2 M EDTA, 0.3 mM 5,5′-dithiobis-(2-nitrobenzoic acid), 0.4 mM NADPH, and 1 U/mL glutathione reductase. Absorbance was recorded at 405 nm every 15 s for 2.5 min. For oxidized glutathione determination, equal volumes of sample supernatant and 2-vinylpyridine were mixed and incubated for 1 h before addition of the reaction mixture. Absorbance was then recorded at 405 nm every 30 s for 5 min. Reduced GSH was calculated from total glutathione and GSSG values.

GPx activity was determined in neuronal lysates using a commercial assay kit (cat. no. ab102530; Abcam), following the manufacturer’s instructions. Results were normalized to protein content and expressed relative to the untreated control condition. H_2_O_2_ was used at 50 µM for 1 h. LEFH was not evaluated together with H_2_O_2_ or Aβ_1–42_ in these assays.

### 4.13. Assessment of AMPKα Phosphorylation

Primary hippocampal cultures were treated with the indicated lipid extracts, Aβ_1–42_ preparation, or their corresponding combinations. For AMPK inhibition experiments, cells were preincubated with Compound **C** (10 µM; cat. no. P5499) for 30 min before LEFH treatment. Compound **C** remained present during the subsequent treatment period. AMPKα phosphorylated at Thr172 was quantified using a phospho-AMPKα ELISA kit (cat. no. KHO0651; Thermo Fisher Scientific), according to the manufacturer’s protocol. Samples from all experimental conditions were processed in parallel and normalized to the same amount of protein. Because the assay quantified AMPKα Thr172 phosphorylation rather than catalytic kinase activity, the results were interpreted as an indicator of AMPKα activation status and not as a direct measurement of AMPK enzymatic activity [80].

### 4.14. RNA Extraction and Quantitative Real-Time PCR

Total RNA was extracted from primary hippocampal neuronal cultures using TRIzol reagent (Thermo Fisher Scientific, Waltham, MA, USA), according to the manufacturer’s instructions. RNA concentration and purity were assessed by measuring absorbance at 260 and 280 nm, and RNA integrity was evaluated by denaturing agarose gel electrophoresis. Complementary DNA was synthesized from 500 ng of total RNA using SuperScript IV reverse transcriptase and random primers. Quantitative real-time PCR was performed using SYBR Green PCR Master Mix (Thermo Fisher Scientific, Waltham, MA, USA). The amplification protocol consisted of an initial denaturation step at 95 °C for 20 s, followed by 40 cycles of 95 °C for 5 s and 60 °C for 30 s. A melting-curve analysis from 55 to 95 °C was performed to verify amplification specificity. The following primer pairs were used: Slc2a1/GLUT1 Forward: 5′-ATGGATCCCAGCAGCAAGAAG-3′ Reverse: 5′-AGAGACCAAAGCGTGGTGAG-3′ GLUT3 Forward: 5′-GGATCCCTTGTCCTTCTGCTT-3′ Reverse: 5′-ACCAGTTCCCAATGCACACA-3′ GLUT4 Forward: 5′-CGGCTCTGACGATGGGGAA-3′ Reverse: 5′-TTGTGGGATGGAATCCGGTC-3′ Cyclin D1 Forward: 5′-AAAATGCCAGAGGCGGATGA-3′ Reverse: 5′-GCAGTCCGGGTCACACTTG-3′ *Myc* Forward: 5′-GGAGTGGTTCAGGATTGGGG-3′ Reverse: 5′-GGGTAGCTTACCAGAGTCGC-3′ Il6 Forward: 5′-CAACGATGATGCACTTGCAGA-3′ Reverse: 5′-GTGACTCCAGCTTATCTCTTGGT-3′ β-catenin Forward: 5′-TCCTTCACGCAAGAGCAAGTA-3′ Reverse: 5′-CGCACCATGCAGAATACAAATGA-3′ *Tnf*-α Forward: 5′-TGATCGGTCCCCAAAGGGAT-3′ Reverse: 5′-TGTCTTTGAGATCCATGCCGT-3′, ACOX1 Forward: 5′-GCCATTCGATACAGTGCTGTGAG-3′, Reverse: 5′-CCGAGAAAGTGGAAGGCATAGG-3′. PGC-1α Forward: 5′-AGCCGTGACCACTGACAACGAG-3′ Reverse: 5′-GCTGCATGGTTCTGAGTGCTAAG-3′ Cyclophilin A Forward: 5′-TGGAGATGAATCTGTAGGAGGAG-3′ Reverse: 5′-TACCACATCCATGCCCTCTAGAA-3′. Cyclophilin A was selected based on its previous use in this neuronal experimental system. Relative expression was calculated using the 2^−^ΔΔCt method, and each transcript was treated as a prespecified exploratory endpoint [81].

### 4.15. Statistical Analysis

The number of independent biological replicates varied according to the experimental assay and is indicated in the corresponding figure legends. Technical replicates, where applicable, were averaged before statistical analysis so that each independent biological preparation contributed a single value. For hippocampal-slice experiments, each independent tissue preparation was considered a biological replicate; when multiple slices obtained from the same animal were analyzed under the same condition, their values were averaged so that each animal contributed only one independent value. Data are presented as mean ± standard deviation.

Normality and homogeneity of variance were evaluated using the Shapiro–Wilk and Levene tests, respectively. Comparisons among experimental treatment groups at individual endpoints were performed using one-way analysis of variance (ANOVA). The glucose-uptake time-course experiment was analyzed using two-way ANOVA considering treatment and time as factors, including the treatment × time interaction. Significant omnibus ANOVA results were followed by Bonferroni-adjusted multiple comparisons. Glucose-uptake concentration–response curves were fitted by nonlinear regression to the Michaelis–Menten equation, and the resulting apparent Vmax and Km parameters were compared among experimental groups by one-way ANOVA. Statistical significance was defined as *p* < 0.05. The corresponding F statistics, degrees of freedom, and omnibus *p* values are reported in Supplementary Table S2, whereas Bonferroni-adjusted post hoc *p* values are provided in Supplementary Table S3.

## 5. Conclusions

At 0.1 µg/mL, LEFH produced the largest biological effects among the three extracts evaluated and attenuated Aβ_1–42_-associated neuronal damage. LEFH preserved neuronal glucose metabolism under amyloidogenic stress by maintaining glucose uptake, glycolytic flux, and pentose phosphate pathway activity and was associated with a higher apparent maximal glucose uptake capacity without evidence of altered apparent glucose affinity. It also improved cellular energy status and was associated with increased AMPKα Thr172 phosphorylation and *Ppargc1a* expression, supporting the engagement of an AMPK-associated metabolic response without establishing causal pathway dependency. Furthermore, LEFH increased basal GSH content and GPx activity and selectively modulated metabolism- and signaling-associated transcripts. The preservation of glucose uptake and ATP/ADP balance was reproduced in ex vivo hippocampal slices, strengthening the biological relevance of the neuronal findings. Nevertheless, further chemical characterization, mechanistic validation, pharmacokinetic assessment, and in vivo evaluation are required before the translational potential of LEFH can be established.

## Data Availability

The original contributions presented in this study are included in the article and Supplementary Material. Further inquiries can be directed to the corresponding author.

## Supplementary Materials

The following supporting information can be downloaded at: https://www.mdpi.com/article/10.3390/ph19091489/s1, Table S1: Fatty-acid profile of *Macrocystis pyrifera* fronds, stipes, and holdfasts collected in the Magellan region, Chile; Table S2: Selected Bonferroni-adjusted pairwise comparisons for the experimental outcomes; Table S3: Omnibus ANOVA results for the experimental outcomes.

## Abbreviations

The following abbreviations are used in this manuscript:

Aβ	Amyloid-β peptide
AD	Alzheimer’s disease
AMPK	AMP-activated protein kinase
BHT	Butylated hydroxytoluene
DMSO	Dimethyl sulfoxide
2-DG	2-deoxy-D-glucose
FAME	Fatty acid methyl ester
GC-FID	Gas chromatography with flame ionization detection
GLUT1	Glucose transporter 1
G6PDH	Glucose-6-phosphate dehydrogenase
GSH	Glutathione
GPx	Glutathione peroxidase
Hk	Hexokinase
LEFF	Frond-derived lipid extract
LEFS	Stipe-derived lipid extract
LEFH	Holdfast-derived lipid extract
NADPH	Reduced nicotinamide adenine dinucleotide phosphate
PPP	Pentose phosphate pathway
ROS	Reactive oxygen species
