# Supplementary material for: Holdfast-Derived Lipid Extract from the Patagonian Macroalga Macrocystis pyrifera Attenuates Amyloid-β-Induced Neuronal Metabolic Dysfunction In Vitro and Ex Vivo
Source: Pharmaceuticals (Basel). 2026 Sep 18;19(9):1489. doi: 10.3390/ph19091489 (PMC13610707; doi:10.3390/ph19091489)
Supplement: Supplementary file 1 [file pharmaceuticals-19-01489-s001.zip › pharmaceuticals-4541478-supplementary.pdf]

Supplementary Table S1. Fatty-acid profile of Macrocystis pyrifera fronds, stipes, and holdfasts collected in the Magellan region, Chile. Values are expressed as percentages of total identified fatty acids and presented as mean ± SD from three independent extractions per anatomical structure (n = 3 per structure). Data were reanalyzed from the GC-FID dataset previously reported by Méndez et al. [36]. Total n-3 and n-6 fatty acids were calculated separately for each independent extraction, after which the corresponding means and SDs were calculated from the sample-level totals. (−), not detected.

**Fatty acids**	**Fatty-acid notation**	**Percentage fronds (mean ± SD)**	**Percentage stipes (mean ± SD)**	**Percentage holdfast (mean ± SD)**
Tridecanoic acid	C13:0	1.26 ± 0.01	-	-
Myristic acid	C14:0	11.50 ± 0.05	14.55 ± 0.06	14.54 ± 0.12
Pentadecanoic acid	C15:0	1.02 ± 0.34	-	0.24 ± 0.34
Palmitic acid	C16:0	20.82 ± 0.09	18.55 ± 0.06	20.74 ± 0.16
Palmitoleic acid	C16:1*n*-7	2.82 ± 0.01	2.69 ± 0.01	3.99 ± 0.01
Stearic acid	C18:0	0.56 ± 0.01	1.04 ± 0.02	1.28 ± 0.02
Oleic acid	C18:1*n*-9c	11.44 ± 0.06	22.03 ± 0.05	23.27 ± 0.18
Linolelaidic acid	C18:2*n*-6t	0.21 ± 0.30	-	-
Linoleic acid	C18:2*n*-6c	4.33 ± 0.01	4.30 ± 0.06	4.45 ± 0.04
Arachidic acid	C20:0	0.63 ± 0.00	0.88 ± 0.02	1.05 ± 0.00
α-Linolenic acid	C18:3*n*-3	5.69 ± 0.03	3.35 ± 0.01	2.23 ± 0.04
Octadecatetraenoic acid	C18:4*n*-3	9.42 ± 0.04	3.80 ± 0.06	2.32 ± 0.02
γ-Linolenic acid	C18:3*n*-6	-	-	-
8,11,14-cis-Eicosatrienoic acid	C20:3*n*-6	0.79 ± 0.01	1.40 ± 0.13	1.45 ± 0.16
Arachidonic acid	C20:4*n*-6	0.63 ± 0.02	1.33 ± 0.02	0.69 ± 0.12
5,8,11,14,17-cis- Eicosapentaenoic acid	C20:5*n*-3	10.13 ± 0.02	7.10 ± 0.10	5.99 ± 0.06
Eicosatetraenoic acid	C20:4*n*-3	1.18 ± 0.03	0.72 ± 0.05	1.12 ± 0.10
11-cis Eicosanoic acid	C20:1*n*-9	-	-	-
Erucic acid	C22:1*n*-9	12.13 ± 0.03	18.27 ± 0.04	14.25 ± 0.04
Total n-3 fatty acids.	Σ*n*-3	26.42 ± 4.57	14.97 ± 2.73	11.65 ± 2.16
Total n-6 fatty acids.	Σ*n*-6	5.96 ± 1.57	7.02 ± 1.59	6.59 ± 1.64

Supplementary Table S2. Selected Bonferroni-adjusted pairwise comparisons for the experimental outcomes. Adjusted p values are reported. Statistical significance was defined as $p < 0.05$.

Variables	Comparison of treatment	p-value
Cell Viability	CONTROL-Aβ_{1-42}	<0.001*
	CONTROL-LEFF	0.100
	CONTROL-LEFS	1.000
	CONTROL-LEFH	1.000
	Aβ_{1-42}-Aβ_{1-42}+LEFF	0.039*
	Aβ_{1-42}-Aβ_{1-42}+ LEFS	1.000
	Aβ_{1-42}-Aβ_{1-42}+ LEFH	<0.001*
[^{3}H]-2-deoxy-D-glucose uptake in hippocampal neurons	CONTROL-LEFF	<0.001*
	CONTROL- LEFS	1.000
	CONTROL- LEFH	<0.001*
	LEFF - LEFS	<0.001*
	LEFF - LEFH	<0.001*
	LEFS - LEFH	<0.001*
Vmax	CONTROL- LEFF	1.000
	CONTROL- LEFS	1.000
	CONTROL- LEFH	0.063
	LEFF - LEFS	1.000
	LEFF - LEFH	0.095
	LEFS - LEFH	0.022*
Km	CONTROL- LEFF	1.000
	CONTROL- LEFS	1.000
	CONTROL- LEFH	1.000
	LEFF - LEFS	1.000
	LEFF - LEFH	1.000
	LEFS - LEFH	1.000
[^{3}H]-2-deoxy-D-glucose uptake in hippocampal neurons	CONTROL-Aβ_{1-42}	<0.001*
	Aβ_{1-42}-Aβ_{1-42}+ LEFF	0.946
	Aβ_{1-42}-Aβ_{1-42}+ LEFS	1.000
	Aβ_{1-42}-Aβ_{1-42}+ LEFH	<0.001*
Glycolysis rate	CONTROL-Aβ_{1-42}	<0.001*
	CONTROL-LEFF	0.775
	CONTROL-LEFS	1.000
	CONTROL-LEFH	<0.001*
	Aβ_{1-42}-Aβ_{1-42}+LEFF	1.000
	Aβ_{1-42}-Aβ_{1-42}+ LEFS	1.000
	Aβ_{1-42}-Aβ_{1-42}+ LEFH	<0.001*
PPP	CONTROL-Aβ_{1-42}	0.004*
	CONTROL-LEFF	1.000
	CONTROL-LEFS	1.000
	CONTROL-LEFH	1.000
	Aβ_{1-42}-Aβ_{1-42}+LEFF	1.000
	Aβ_{1-42}-Aβ_{1-42}+ LEFS	0.183

	$A\beta_{1-42}$-$A\beta_{1-42}$+ LEFH	0.028*
GSH levels	CONTROL- LEFF	1.000
	CONTROL- LEFS	0.799
	CONTROL- LEFH	<0.001*
	CONTROL- H_2O_2	<0.001*
	LEFF - LEFS	1.000
	LEFF - LEFH	<0.001*
	LEFF- H_2O_2	<0.001*
	LEFS - LEFH	<0.001*
	LEFS- H_2O_2	<0.001*
	LEFH- H_2O_2	<0.001*
GPx activity	CONTROL- LEFF	1.000
	CONTROL- LEFS	1.000
	CONTROL- LEFH	0.001*
	CONTROL- H_2O_2	<0.001*
	LEFF - LEFS	1.000
	LEFF - LEFH	<0.001*
	LEFF- H_2O_2	<0.001*
	LEFS - LEFH	<0.001*
	LEFS- H_2O_2	<0.001*
	LEFH- H_2O_2	<0.001*
AMPK activation status	CONTROL-$A\beta_{1-42}$	<0.001*
	CONTROL- LEFF	1.000
	CONTROL- LEFS	1.000
	CONTROL- LEFH	$p < 0.001$
	$A\beta_{1-42}$-$A\beta_{1-42}$+ LEFF	1.000
	$A\beta_{1-42}$-$A\beta_{1-42}$+ LEFS	1.000
	$A\beta_{1-42}$-$A\beta_{1-42}$+ LEFH	0.002*
	CC versus CC + LEFH	$p < 0.001$
ATP/ADP ratio	CONTROL- LEFF	1.000
	CONTROL- LEFS	0.240
	CONTROL- LEFH	$p < 0.001$
	LEFF - LEFS	0.013*
	LEFF - LEFH	$p < 0.001$
	LEFS - LEFH	$p < 0.001$
PGC-1α	CONTROL- LEFF	1.000
	CONTROL- LEFS	1.000
	CONTROL- LEFH	$p < 0.001$
	LEFF - LEFS	1.000
	LEFF - LEFH	$p < 0.001$
	LEFS - LEFH	$p < 0.001$
ACOX1	CONTROL- LEFF	1.000
	CONTROL- LEFS	1.000
	CONTROL- LEFH	$p < 0.001$
	LEFF - LEFS	1.000
	LEFF - LEFH	$p < 0.001$
	LEFS - LEFH	$p < 0.001$
TNF-α	CONTROL- LEFF	1.000
	CONTROL- LEFS	1.000

	CONTROL- LEFH	0.220
	LEFF - LEFS	1.000
	LEFF - LEFH	0.858
	LEFS - LEFH	0.190
Il6 mRNA expression	CONTROL- LEFF	1.000
	CONTROL- LEFS	0.030*
	CONTROL- LEFH	$p < 0.001$
	LEFF - LEFS	0.003*
	LEFF - LEFH	$p < 0.001$
	LEFS - LEFH	$p < 0.001$
GLUT 1	CONTROL- LEFF	1.000
	CONTROL- LEFS	1.000
	CONTROL- LEFH	1.000
	LEFF - LEFS	0.774
	LEFF - LEFH	1.000
	LEFS - LEFH	1.000
GLUT 3	CONTROL- LEFF	0.381
	CONTROL- LEFS	1.000
	CONTROL- LEFH	1.000
	LEFF - LEFS	0.449
	LEFF - LEFH	0.134
	LEFS - LEFH	1.000
GLUT 4	CONTROL- LEFF	1.000
	CONTROL- LEFS	1.000
	CONTROL- LEFH	$p < 0.001$
	LEFF - LEFS	1.000
	LEFF - LEFH	$p < 0.001$
	LEFS - LEFH	$p < 0.001$
c-Myc	CONTROL- LEFF	1.000
	CONTROL- LEFS	1.000
	CONTROL- LEFH	$p < 0.001$
	LEFF - LEFS	1.000
	LEFF - LEFH	$p < 0.001$
	LEFS - LEFH	$p < 0.001$
Cyclin-D1	CONTROL- LEFF	1.000
	CONTROL- LEFS	1.000
	CONTROL- LEFH	$p < 0.001$
	LEFF - LEFS	1.000
	LEFF - LEFH	$p < 0.001$
	LEFS - LEFH	$p < 0.001$
β-Catenin	CONTROL- LEFF	1.000
	CONTROL- LEFS	1.000
	CONTROL- LEFH	$p < 0.001$
	LEFF - LEFS	1.000
	LEFF - LEFH	$p < 0.001$
	LEFS - LEFH	$p < 0.001$
Glucose uptake in hippocampal slice	CONTROL-$A\beta_{1-42}$	$p < 0.001$
	CONTROL- LEFF	1.000

	CONTROL- LEFS	1.000
	CONTROL- LEFH	$p < 0.001$
	$A\beta_{1-42}$-$A\beta_{1-42}$+ LEFF	1.000
	$A\beta_{1-42}$-$A\beta_{1-42}$+ LEFS	1.000
	$A\beta_{1-42}$-$A\beta_{1-42}$+ LEFH	0.005*
ATP/ADP ratio	CONTROL-$A\beta_{1-42}$	0.023*
	CONTROL- LEFF	1.000
	CONTROL- LEFS	0.638
	CONTROL- LEFH	$p < 0.001$
	$A\beta_{1-42}$-$A\beta_{1-42}$+ LEFF	1.000
	$A\beta_{1-42}$-$A\beta_{1-42}$+ LEFS	1.000
	$A\beta_{1-42}$-$A\beta_{1-42}$+ LEFH	$p < 0.001$

(*) Indicates significant values at a confidence level of 95% ($p<0.05$).

Supplementary Table S3. Omnibus ANOVA results for the experimental outcomes. F statistics, treatment and error degrees of freedom, and omnibus p values are reported for the corresponding analyses. Statistical significance was defined as $p < 0.05$. df, degrees of freedom; F, F statistic.

**Variables**	**Factor**	**df**	**F**	**p-value**
Cell Viability	Treatment	7	57.15	<0.001*
	Error	32		
[^{3}H]-2-deoxy-D-glucose uptake in hippocampal neurons	Time	7	712.318	<0.001*
	Treatment	3	159.921	<0.001*
	Time x Treatment	21	17.836	<0.001*
	Error	96		
Vmax	Treatment	3	6.593	0.019
	Error	7		
Km	Treatment	3	0.431	0.737
	Error	7		
[^{3}H]-2-deoxy-D-glucose uptake in hippocampal neurons	Treatment	4	92.69	<0.001*
	Error	20		
Glycolysis rate	Treatment	7	57.17	<0.001
	Error	32		
PPP	Treatment	7	4.69	<0.001
	Error	32		
GSH levels	Treatment	4	147.862	<0.001
	Error	25		
GPx activity	Treatment	4	131.349	<0.001
	Error	19		
AMPK activation status	Treatment	7	61.34	<0.001
	Error	32		
ATP/ADP ratio	Treatment	3	33.735	<0.001
	Error	16		
PGC-1α	Treatment	3	32.283	<0.001
	Error	16		
ACOX1	Treatment	3	30.798	<0.001
	Error	15		
TNF-α	Treatment	3	2.388	0.107
	Error	16		
IL-6	Treatment	3	42.690	<0.001
	Error	15		
GLUT 1	Treatment	3	0.935	0.447
	Error	16		
GLUT 3	Treatment	3	2.448	0.101
	Error	16		
GLUT 4	Treatment	3	435.857	<0.001
	Error	16		
c-Myc	Treatment	3	37.147	<0.001
	Error	16		
Cyclin-D1	Treatment	3	39.691	<0.001
	Error	16		
β-Catenin	Treatment	3	35.750	<0.001
	Error	16		

Glucose uptake in hippocampal slice	Treatment	7	64.312	<0.001
	Error	32		
ATP/ADP ratio	Treatment	7	50.988	<0.001
	Error	32		

(*) Indicates significant values at a confidence level of 95% ($p<0.05$).
